# Key performance indicators for hospital clinical pharmacy services: results of a global Delphi study

**DOI:** 10.1007/s11096-026-02126-y

**Published:** 2026-04-01

**Authors:** Lucas Magedanz, Dayani Galato, Clinical Pharmacy KPI research team, Arash Apornak, Brian Magennis, Chahnez Charfi Triki, Chigozie Gloria Anene-Okeke, Craig J. Beavers, Douglas Doucette, Eric Pitters, Georgia F. Lloyd, Glenys Davidson, Hugo Lopes, Iga Pawłowska, Iria Miguens Blanco, Jerome Ng, JIthangi Wanigasinghe, Julio Burman, Kent Toombs, Lécio Figueira Pinto, Lela Sturua, Leonor Periañez Parraga, Lilia Nuñez Orozco, Lira Margarita Tatis Caraballo, Lisa Todd, María Martín Cerezuela, Maria Theodoridou, Marina Dorofeeva, Martin Luke Canning, Meghdad Rahati, Michelle van der Merwe, Mireia Puig-Campmany, Natalia Chirun, Nicole M. Acquisto, Olavo Fernandes, Olivia Dalleur, Philip K. King, Richard S. Slavik, Santiago Tomás Vecina, Shaka Roberts, Steve Shalansky, Tiago Costa, Ton de Leeuw, Vasiliki Kapaki, Vladimir Donev, William M. Semchuk, Fernando Fernandez-Llimos

**Affiliations:** 1 University of Brasília, Brasília, DF Brazil; 2 UCIBIO–Applied Molecular Biosciences Unit, i4HB–Institute for Health and Bioeconomy, Laboratory of Pharmacology, Faculty of Pharmacy, University of Porto, Porto, Portugal

**Keywords:** Delphi technique, Outcome and process assessment, health care, Pharmacy service, hospital, Quality assurance, health care, Quality indicators, health care

## Abstract

**Introduction:**

The contribution of clinical pharmacy services (CPS) to patient outcomes and system performance is still not consistently measured. Most key performance indicators (KPIs) have been suggested only by pharmacists, without formal piloting or validation, and without benchmarking reference values. KPIs are indicators used to quantify pharmacists’ clinical activities and their association with improvements in patient safety, therapeutic outcomes, and healthcare costs. There is a need for a globally informed, multi-stakeholder consensus on a core set of KPIs for hospital clinical pharmacy services that are explicitly outcome-based and suitable for international use.

**Aim:**

This study aimed to reach a consensus on a core set of KPIs for clinical pharmacy activities in hospital settings, achieved through a multi-stakeholder consensus process.

**Method:**

The 3-round Delphi technique was used to reach a consensus. Panellists were systematically selected from three groups of stakeholders: ‘Clinical Pharmacy KPI Experts’, ‘Healthcare Systems Assessment Experts’, and ‘Patient Association Representatives’. The initial set of KPIs was based on themes identified in a previous review study. During the three rounds, the consensus thresholds increased from 60 to 70% and then to 80%. Panellists could suggest new KPIs and propose modifications to those already suggested.

**Results:**

Of the 480 potential experts initially identified, 342 valid email addresses were found, of which 49 (from 25 countries) committed to the Delphi study, including 22 clinical pharmacy experts, 21 patient representatives, and six healthcare systems assessment experts. A total of 43 panellists from 23 different countries completed the third round. Starting with 12 KPIs (plus one added in the first round), the panellists agreed on five KPIs, which were distributed as follows: Three clinical KPIs (adverse drug event rate, medication-related near miss event rate and medication goal achievement rate); one economic KPI (average optimised treatment cost difference); and one humanistic outcome KPI (patient satisfaction survey results). A manual containing the KPI information sheets was created.

**Conclusion:**

A global panel of stakeholders, including pharmacists, other healthcare professionals, and patient representatives, agreed on a core set of KPIs for hospital-provided CPS. This set comprises five measures covering three types of outcomes: economic, clinical, and humanistic.

**Supplementary Information:**

The online version contains supplementary material available at 10.1007/s11096-026-02126-y.

## Impact statements

- The study established a core set of key performance indicators (KPIs) for hospital-based clinical pharmacy services. These KPIs align with Donabedian's definition of outcomes, which is based on evaluating the effects or results of healthcare interventions or services on patients' health status or well-being.
- The agreed KPIs included a total of five measures: three focused on clinical outcomes, one on economic aspects and one on humanistic factors.
- A core set of KPIs can facilitate more informed decision-making, optimise resource allocation and drive policy development within the healthcare sector. Furthermore, these standardised KPIs allow for comparisons between clinical pharmacy services (CPS) at different institutions and help to establish consistent benchmarks.

## Introduction

Clinical pharmacy services (CPS) have become integral to hospital care, yet their contribution to patient outcomes and system performance remains inconsistently measured. Key performance indicators (KPIs) are widely recognized as essential tools for monitoring the quality, efficiency, and impact of health services, but implementation guidelines for hospital clinical pharmacy services rarely specify robust, standardized measures [1, 2]. Previous initiatives have proposed numerous indicators; however, they are often locally developed, predominantly process-oriented, and methodologically weak, which limits their use for accountability, benchmarking, and strategic decision-making [3–5].

In a recent systematic review, 225 KPIs for hospital clinical pharmacy services were identified across 13 studies, revealing major gaps in both content and quality [6]. Less than 6% of these indicators were classified as outcome measures within Donabedian’s structure–process–outcome framework [7]. Most KPIs had been suggested by pharmacists, frequently without formal piloting or validation, and none of the studies provided benchmarking reference values, which severely constrains the capacity to compare services across institutions or jurisdictions. This systematic review highlighted the urgent need for methodologically sound, outcome-oriented KPIs that can capture the real impact of hospital clinical pharmacy services on patients and health systems.

Beyond the imbalance towards process measures, existing KPI sets often underrepresent the perspectives of key stakeholders such as patients, managers, and non-pharmacist clinicians. Earlier Delphi-based efforts and national exercises to define clinical pharmacy KPIs have primarily recruited pharmacists, with limited inclusion of other professional groups and virtually no systematic involvement of patient representatives [8]. As a result, many proposed indicators focus on documenting professional activities rather than on quantifiable improvements in patient health, experience, or costs, which weakens their relevance for value-based healthcare and resource allocation [9].

There is increasing consensus that performance evaluation in healthcare should be explicitly patient-centred and outcome-driven [10], aligning measures with the three components of the ECHO model: clinical, economic, and humanistic outcomes [11]. For hospital clinical pharmacy services, this implies prioritizing indicators that reflect changes in adverse drug events, readmissions, treatment costs, and patient-reported experiences, rather than simply counting interventions or documenting workflow [12, 13]. However, the scarcity of rigorously developed, outcome-oriented KPIs, combined with the lack of a globally agreed core set, prevents meaningful comparison between services and hinders the development of policies that recognize and reward their contribution.

In this context, there is a clear need for a globally informed, multi-stakeholder consensus process to define a core set of KPIs for hospital clinical pharmacy services that are explicitly outcome-based and suitable for international use. By integrating perspectives from clinical experts, healthcare systems assessment specialists, and patient representatives, such an approach can generate indicators that are both methodologically robust and perceived as relevant by those who deliver, manage, and receive care.

### Aim

The aim of this study was to reach consensus on a core set of KPIs for clinical pharmacy activities in hospital settings through a multi-stakeholder consensus process.

## Method

### Study design

The Delphi technique was used to reach a consensus. This approach is particularly well-suited to international applications, as it does not require face-to-face meetings. It is often used to develop guidelines and policies in areas where evidence is limited [14, 15]. The study protocol was previously published on the Open Science Framework (OSF) and is available at 10.17605/OSF.IO/ENYGT.

### Delphi panel identification and recruitment

Three distinct groups of stakeholders were identified: (i) ‘Clinical Pharmacy KPI Experts’, who are experienced in developing and implementing clinical pharmacy KPIs in hospital settings; (ii) ‘Healthcare Systems Assessment Experts’, who are experts in evaluating healthcare services within hospital environments; (iii) ‘Patient Association Representatives’, who are representatives of patient association organisations. A specific panellist identification strategy was designed for each group, which is detailed in Supplementary File 1. The goal was to recruit 40 experts, considering a 66% retention rate across rounds two and three, in line with recommendations for healthcare quality indicator Delphi studies [16–19].

Initial contact with potential panellists was made via email by a member of the study steering committee. The invitation provided comprehensive information about the study’s objectives, scope and expected outcomes, as well as the required timeline for participation. No financial compensation or remuneration was offered at any point. Participation in the study depended on the panellist confirming their commitment.

### Key performance indicators development

The initial set of KPIs was developed by the steering committee, who based them on themes that had been identified in a previous review study of the literature [6]. A key requirement was that the KPIs should not be specific to a particular drug or disease. The indicators adhered to Donabedian’s definition of outcomes as ‘measures that assess the effects or results of healthcare interventions or services on patients’ health status or well-being’ [20]. Furthermore, the KPIs were designed to cover all three categories of Kozma’s model: clinical, economic and humanistic outcomes [11]. Combining Donabedian’s SPO framework with Kozma’s ECHO model is a common practice in health services research to link the quality of healthcare delivery, measured by patients’ outcomes, to a multidimensional outcome set (clinical, economic, and humanistic), thus enabling a more comprehensive evaluation of healthcare interventions.

Each KPI was given a title, defined its purpose, set out its calculation formula and specified its outcome measure. To provide panellists with in-depth information, information sheets containing key concepts, examples of use and operational considerations (such as risk adjustment variations) were written. These documents were made available to panellists via a link in the online questionnaire. The Steering Committee was responsible for developing and updating all KPI materials.

### Delphi survey

The three-round Delphi survey was conducted online between February and April 2024 using REDCap (Research Electronic Data Capture), a secure, web-based tool for capturing data in research studies (https://redcapbrasil.com.br). The Steering Committee pilot-tested the online questionnaire. The survey was conducted exclusively in English.

For each round, panellists were given up to three weeks to evaluate the proposed KPIs. To encourage participation, weekly reminders were sent to non-respondents, increasing in frequency to daily reminders during the final five days of each round. Panellists rated the relevance of the KPIs using a three-point scale. Initially, the options were ‘yes’, ‘maybe’ and ‘no’. After the first round, the ‘maybe’ option was changed to ‘I don’t know’ to avoid implicit acceptance of the original term. Comment boxes were made available for each KPI, allowing panellists to suggest improvements and additional indicators.

The consensus thresholds were predetermined and increased progressively across the three rounds: 60% in the first round, 70% in the second, and 80% in the third round. A KPI advanced to the next round if the percentage of ‘Yes’ responses met or surpassed the threshold for that round. Additionally, a KPI could advance even if the consensus threshold was not achieved, provided that the Steering Committee approved significant modifications to the KPI’s title, purpose, or calculation formula as suggested by the panellists. This strategy was selected based on a double rationale:

- Preventing premature attrition (sensitivity): In the initial rounds, priority was inclusivity. Setting a lower consensus threshold (60%) in Round 1 ensured that the technique would not prematurely discard KPIs that might have significant clinical value but required further refinement or participant reflection.
- Enhancing stringency and rigor (specificity): As the rounds progressed, the objective shifted from exploration to validation, by sequentially increasing the threshold (80%).

After each round, the panellists received a personalised report comparing their responses to the overall consensus rating. This report also outlined any KPI improvements that had been accepted and implemented for the next round. Only panellists who had participated in the previous round received the report and were eligible to participate in the next one. Additional information was collected. In the first round, the panellists provided details about their professional and personal backgrounds. Country affiliation was determined by panellists’ affiliations during recruitment or the locations of their associated patient organisations.

The data from the Delphi survey were analysed in Microsoft Excel to produce descriptive statistics, including frequencies and percentages, to establish KPI consensus. Additionally, exploratory Fisher’s exact test was performed using IBM SPSS Statistics v.29 to evaluate potential differences in KPI evaluations between healthcare experts (clinical pharmacy experts and healthcare systems assessment experts) and patient association representatives. This test was chosen for its suitability in analysing categorical data, especially when expected frequencies in contingency table cells are small [21]. A *p*-value of less than 0.05 was set as the threshold for statistical significance.

### Ethics approval

This study was conducted following the Ethical Guidelines for Internet-Mediated Research [22], and reported following DELPHISTAR recommendations [23]. Informed consent was obtained from all individual participants. Ethical approval was obtained from the University of Brasília Ethics Committee (registration number 6.280.723).

## Results

### Expert panel member recruitment and participation

An initial screening of 480 potential experts yielded 342 valid email addresses, all of them were contacted and 49 committed to the Delphi study. The distribution of participants comprised 22 clinical pharmacy experts, 21 patient association representatives and six healthcare systems assessment experts. The study maintained a high retention rate across its three rounds. In the first round, 47 out of 49 participants completed the survey, giving a response rate of 95.9%. In the second round, 43 out of 47 panellists responded (91.5%), and there were no drop-outs in the third round (100%). The Delphi survey’s overall retention rate was 87.8%.

Of the panellists who participated in all rounds, 81.4% reported holding a degree in healthcare, 67.4% had experience in healthcare management and 88.4% had been hospitalised, which gave them also the patient’s perspective. The 43 participants were geographically distributed across all continents, with 16 from Europe, 14 from the Americas, five from Asia, five from Oceania and three from Africa (Supplementary File 2). Table 1 provides further details on the panellists’ professional experience, categorised by group.

**Table 1:** Experience characteristics of Delphi panellists

	Round 1 (n = 47)			Rounds 2 and 3 (n = 43)		
Variable/Group	Clinical Pharmacy experts (n = 21)	Healthcare systems assessment experts (n = 6)	Patient Associations representatives (n = 20)	Clinical Pharmacy experts (n = 21)	Healthcare systems assessment experts (n = 4)	Patient Associations representatives (n = 18)
Field of experience*, n (%)						
Academy/Professor	9 (43)	3 (50)	–	9 (43)	2 (50)	–
Manager/Director	6 (29)	2 (33)	–	6 (29)	2 (50)	–
Medicine	–	2 (33)	1 (5)	–	–	1 (6)
Nursing	–	–	2 (10)	–	–	2 (11)
Pharmacy	19 (91)	–	1 (5)	19 (91)	–	1 (6)
*Panellists characteristics, n (%)*						
Panellists who declare have a healthcare professional degree	21 (100)	5 (83)	11 (55)	21 (100)	4 (100)	10 (56)
Panellists who declare experience in healthcare management	18 (86)	4 (67)	9 (45)	18 (86)	3 (75)	8 (44)
Panellists who declare experience in being hospitalized	17 (81)	5 (83)	20 (100)	17 (81)	3 (75)	18 (100)

^*^As declared in affiliation details (each panellist can have more than one field of expertise)

### Delphi survey consensus process

In Round 1, consensus was reached on 10 out of 12 KPIs (83.3%). The two KPIs that did not reach the threshold in the first round (C05 and E01) advanced to the next round due to substantial improvements being made after panellists’ suggestions. Enhancements were made to eight KPIs that had achieved consensus in the first round (C02, C04, C06, C07, E02, E03, H01 and H03). One new KPI was introduced based on panellist feedback. In Round 2, 10 out of 13 KPIs (76.9%) reached the consensus threshold of ≥ 70%. Five indicators (C02, C04, C08, E02 and E03) were improved further based on feedback. In the final round, five out of ten KPIs (50%) surpassed the ≥ 80% consensus threshold, resulting in three clinical, one economic and one humanistic KPI. Table 2 shows the consensus rates for each KPI throughout the survey, and Supplementary File 3 details the evolution of the KPIs throughout the Delphi rounds. The final core set of CPS KPIs is presented in Table 3. A manual comprising the KPI information sheets integrating the core set is available in Supplementary File 4.

**Table 2:** KPI consensus rate in each round of the Delphi survey

KPI category	KPI code	KPI title*	Outcome measure*	Round 1 (≥ 60%)	Round 2 (≥ 70%)	Round 3 (≥ 80%)
Clinical	C01	Adverse drug event rate	Incidence rate of ADEs per 1,000 patient days followed by the CPS, in the hospital in a certain period of time	72.3%	93.0%	97.7%
	C02	Medication-related near miss event rate	Incidence rate of near miss events avoided per 1,000 patient-days followed by the CPS, in the hospital in a certain period of time	68.1%	76.7%	93.0%
	C03	Average length of stay	Average days of the length of stay of patients followed by the CPS, in the hospital in a certain period of time	63.8%	67.4%	Not in round 3
	C04	Medication goal achievement rate	Incidence rate of drug therapies that achieved the planned therapeutic goal per 1,000 therapies followed by the CPS, in the hospital in a certain period of time	76.6%	83.7%	81.4%
	C05	Medication-related cancelled planned care rate	Incidence rate of cancelled planned care per 1,000 patient days followed by the CPS, in the hospital in a certain period of time	44.7%	62.8%	Not in round 3
	C06	All-cause unplanned 30-day readmission rate	Incidence rate of all-cause unplanned 30-day readmissions in hospital ward per 1,000 patients followed by the CPS, in the hospital in a certain period of time	68.1%	74.4%	74.4%
	C07	All-cause mortality rate	Incidence rate of deaths per 1,000 admissions in patients followed by the CPS, in the hospital in a certain period of time	63.8%	79.1%	76.7%
	C08	All-cause 30-day emergency department visit rate	Incidence rate of all-cause unplanned 30-day emergency department visit per 1,000 patients followed by the CPS, in the hospital in a certain period of time	Not in round 1	76.7%	72.1%
Economic	E01	Average Clinical Pharmacy Service revenue generated	Estimated average revenue generated (in US dollars or local currency) per 1,000 patient days followed by the CPS, in the hospital in a certain period of time	46.8%	55.8%	Not in round 3
	E02	Total optimized treatment cost difference	Estimated total costs differences (in US dollars or local currency) from all treatments optimized by the CPS, in the hospital in a certain period of time	76.6%	81.4%	74.4%
	E03	Average optimized treatment cost difference	Average of the estimated total costs differences (in US dollars or local currency) from the treatments optimized by the CPS, in the hospital in a certain period of time	68.1%	81.4%	81.4%
Humanistic	H01	Negative Patient Reported Experience Measures (PREMs) rate	Incidence rate of negative PREMs per 1,000 PREMs related to the CPS, in the hospital, in a certain period of time	74.5%	76.7%	74.4%
	H02	Patient satisfaction survey results	Satisfaction perception in patients followed by the CPS in the hospital, in a certain period of time	72.3%	86.0%	93.0%

^*^Shown as the last updated version of each KPI

**Table 3:** Consensus core set of key performance indicators for clinical pharmacy services in hospitals established by the Delphi survey

KPI category	KPI title	Calculation formula	Outcome measure
Clinical	Adverse drug event rate	[(number of adverse drug events (ADEs) that occurred in patients followed by the Clinical Pharmacy Service (CPS), in a certain period of time) ÷ (total number of patient days followed by the CPS in the hospital, in the same period of time)] × 1,000	Incidence rate of ADEs per 1,000 patient days followed by the CPS, in the hospital in a certain period of time
	Medication-related near miss event rate	[(number of suspected medication-related near-miss events that occurred in patients followed by the Clinical Pharmacy Service (CPS), in a certain period of time) ÷ (total number of patient days followed by the CPS in the hospital, in the same period of time)] × 1,000	Incidence rate of near miss events avoided per 1,000 patient-days followed by the CPS, in the hospital in a certain period of time
	Medication goal achievement rate	[(number of medications that achieved the planned goal in patients followed by the Clinical Pharmacy Service (CPS), in a certain period of time) ÷ (total number of drug therapies followed by the CPS in the hospital, in the same period of time)] × 1,000	Incidence rate of drug therapies that achieved the planned therapeutic goal per 1,000 therapies followed by the CPS, in the hospital in a certain period of time
Economic	Average optimized treatment cost difference	∑ (sum) of all "individual" direct treatment cost cases saved by CPS, in a certain period of time: [(cost of treatment day (in US dollars or local currency) of the optimized treatment suggested by the CPS and ACCEPTED by the healthcare team)—(cost of treatment day (in US dollars or local currency) of the given medication (original)) x (number of days that the original medication was replaced by the optimized treatment)] ÷ (number of optimized therapeutic options suggested by the CPS and ACCEPTED by the healthcare team in the hospital, in the same period of time)	Average of the estimated total costs differences (in US dollars or local currency) from the treatments optimized by the CPS, in the hospital in a certain period of time
Humanistic	Patient satisfaction survey results	(Results of satisfaction surveys collected from patients followed by the Clinical Pharmacy Service (CPS) in the hospital, in a certain period of time)	Satisfaction perception in patients followed by the CPS in the hospital, in a certain period of time

Minimal differences in KPI relevance ratings were observed between groups (Table 4). In Round 1, only KPI E01 showed significant variation in acceptance levels (Fisher’s exact test, *p* = 0.016). In Round 2, differences were observed in KPIs C05 and C06 (*p* = 0.023 and *p* = 0.038, respectively). No significant differences in agreement were observed in the final round.

**Table 4:** Comparison of KPI relevance evaluation between healthcare experts and patient association representatives

Category	KPI code	KPI title*	Round 1			Round 2			Round 3		
			Consensus rate			Consensus rate			Consensus rate		
			Healthcare experts** (n = 27)	Patient representatives (n = 20)	*p*-value***	Healthcare experts** (n = 25)	Patient representatives (n = 18)	*p*-value***	Healthcare experts** (n = 25)	Patient representatives (n = 18)	*p*-value***
Clinical	C01	Adverse drug event rate	66.7%	80.0%	0.605	88.0%	100.0%	0.502	96.0%	100.0%	1.000
Clinical	C02	Medication-related near miss event rate	59.3%	80.0%	0.224	68.0%	88.9%	0.170	88.0%	100.0%	0.502
Clinical	C03	Average length of stay	63.0%	65.0%	1.000	76.0%	55.6%	0.359	Not in round 3	Not in round 3	–
Clinical	C04	Medication goal achievement rate	70.4%	85.0%	0.557	84.0%	83.3%	1.000	76.0%	88.9%	0.434
Clinical	C05	Medication-related cancelled planned care rate	33.3%	60.0%	0.103	56.0%	72.2%	0.023***	Not in round 3	Not in round 3	–
Clinical	C06	All-cause unplanned 30-day readmission rate	66.7%	70.0%	0.754	88.0%	55.6%	0.038	80.0%	66.7%	0.628
Clinical	C07	All-cause mortality rate	55.6%	75.0%	0.367	68.0%	94.4%	0.086	72.0%	83.3%	0.677
Clinical	C08	All-cause 30-day emergency department visit rate	Not in round 1	Not in round 1	–	88.0%	61.1%	0.054	80.0%	61.1%	0.369
Economic	E01	Average Clinical Pharmacy Service revenue generated	33.3%	65.0%	0.016***	56.0%	55.6%	0.702	Not in round 3	Not in round 3	–
Economic	E02	Total optimized treatment cost difference	66.7%	90.0%	0.175	76.0%	88.9%	0.358	72.0%	77.8%	0.178
Economic	E03	Average optimized treatment cost difference	66.7%	70.0%	1.000	84.0%	77.8%	0.328	84.0%	77.8%	0.077
Humanistic	H01	Negative Patient Reported Experience Measures (PREMs) rate	70.4%	80.0%	0.243	68.0%	88.9%	0.088	76.0%	72.2%	0.229
Humanistic	H02	Patient satisfaction survey results	70.4%	75.0%	1.000	84.0%	88.9%	1.000	88.0%	100.0%	0.252

^*^Shown as the last updated version of each KPI. **Healthcare experts group consider ‘‘Clinical pharmacy experts’ + ’Healthcare systems assessment experts’. ***Fisher’s exact test

## Discussion

### Statement of key findings

In this study, hospital pharmacists, healthcare leaders and patient representatives agreed on a core set of outcome-driven KPIs for CPS in hospital settings using a global Delphi technique. The final core set comprises five KPIs, including three clinical, one economic and one humanistic KPI. A manual containing the KPI information sheets, along with calculation formulas and operational considerations such as risk adjustment, has also been made available. Together, these KPIs provide a globally comprehensive framework for evaluating CPS performance, serving as a core set that hospitals worldwide can adapt to their specific clinical and institutional contexts.

### Strengths and weaknesses

We conducted a rigorous three-round Delphi survey, recruiting a diverse international stakeholder panel representing all continents. Incorporating stakeholders from all continents enabled the study to gather a variety of expertise, including that of healthcare professionals (pharmacists, physicians and nurses), academic researchers and patient association representatives. Notably, including patients as active participants enhanced the value of the results. This heterogeneous decision-making group was crucial for selecting KPIs that are universally applicable and can be implemented effectively across different healthcare settings worldwide. Furthermore, avoiding a disease- or drug-specific focus ensures that the agreed KPIs are less reliant on specific resources or technologies and are more widely applicable across hospital specialties.

This study has several limitations. Firstly, excluding disease- or drug-specific studies from our eligibility criteria may have limited the variety of KPIs. However, this could also be viewed as a strength, as it promotes the universality of the measures. Secondly, the focus of this Delphi exercise was solely on the relevance of the proposed KPIs, rather than on practical implementation aspects such as feasibility, measurability and practicality. However, we consider these factors to be highly context-dependent and to vary significantly by location or hospital, thus placing them beyond the scope of this research. Thirdly, the initial recruitment rate was relatively low; however, the high retention rate throughout the three rounds ensured a stable and committed expert panel. Furthermore, while the Delphi panel was international, there was a geographic imbalance with limited representation from Africa and parts of Asia. This may affect the immediate generalizability of the findings to these regions. Additionally, as is common in Delphi studies, participants with stronger convictions may have been more inclined to engage. To ensure robust agreement, we adopted a stringent threshold of 80% in the final round. Finally, the subgroup comparisons should be interpreted with caution. These analyses were exploratory and, due to small subgroup sizes, were not powered to detect subtle differences. Consequently, no adjustments for multiple testing were made, as the goal was to identify divergent perspectives rather than confirm statistical hypotheses.

### Interpretation

In an increasingly complex and resource-constrained healthcare environment, all services and technologies, including CPS, must demonstrate their contribution to patient outcomes and system efficiency to ensure their sustainability and value. To achieve this, KPIs should focus on quantifiable improvements in patient care and health outcomes [10]. The KPIs agreed upon in this study address a gap in the literature by proposing a set of KPIs that are fully aligned with outcome-based evaluation. This provides a more accurate and meaningful assessment of the effectiveness of CPS.

Effective stakeholder selection is crucial in consensus studies, ensuring a broad and representative range of perspectives [24, 25]. Despite the growing recognition of the importance of patient involvement in enhancing the overall value of healthcare services [26], previous studies have criticised the excessively pharmacist-centred design of KPIs [6]. The present study not only included a multidisciplinary group of healthcare professionals but also incorporated the patient’s perspective. This was achieved by engaging with representatives from patient associations who are actively involved with healthcare systems and concerned about the quality of care they receive. Our inter-group analysis showed that the patient representatives were as rigorous in their evaluations as the professional stakeholders.

The five KPIs agreed in this study address the three types of health outcome. Of the three KPIs focused on clinical outcomes, one monitors drug effectiveness in patients, while the other two focus on patient safety. Notably, the ‘near-miss’ KPI encompasses a key activity of clinical pharmacists, as highlighted in a study conducted in South Korea involving over 200 hospital pharmacists. In this study, nearly half of the participants (43.9%) identified five or more prescription-related near-miss events per month [27].

Economic evaluations are equally important for demonstrating the sustainability of clinical services within the healthcare system. Previous studies have shown that interventions aimed at optimising medication use can lead to substantial cost savings [28]. One practical example is the calculation of ‘presumed cost avoidance’, which measures the cost difference between the initial therapeutic approach and the changes made following a pharmacist’s intervention, such as adjusting the dose, reducing therapy duration or stopping treatment early, provided that therapeutic effectiveness and safety are maintained [29, 30].

Finally, humanistic KPIs, which evaluate subjective and qualitative aspects of care such as patient satisfaction and health-related quality of life, are becoming increasingly relevant in the context of pharmacist-led clinical services [31]. In our study, the measure ‘Patient satisfaction survey results’ does not specify any instrument. Instead, we recommend that each hospital consider the results of satisfaction surveys already implemented locally.

However, it is important to note that some frequently used metrics, such as mortality rate, length of stay and readmission rate, were not agreed upon in the final round of this study. This may be because some experts believe that these measures do not directly reflect the specific contributions of clinical pharmacists, as they are influenced by factors involving other healthcare professionals [32, 33].

### Further research

Although consensus-based approaches offer a lower level of evidence than experimental designs, they are widely accepted for addressing gaps in areas of science that still need to be explored, particularly in the creation of core outcome sets. Future studies are essential to evaluate the operational aspects of the proposed KPIs, i.e. their feasibility, implementability and real-world adaptability. Additionally, qualitative research is important in exploring how stakeholders understand and interpret the core set KPIs. Finally, establishing benchmarks based on these KPIs will be crucial in defining minimum quality standards and allowing comparisons to be made between the impact of CPS in different healthcare environments.

## Conclusion

The proposed core set of KPIs comprises five measures: Adverse drug event rate; medication-related near miss event rate; medication goal achievement rate; average optimised treatment cost difference; and patient satisfaction survey results. These indicators represent a significant step towards establishing an internationally recognised core set of KPIs for measuring CPS performance. Thus, we believe that this core set could lead to better-informed decision-making, resource allocation and policy development in healthcare, thereby serving as a starting point for further validation to raise care standards and encourage continuous improvement in CPS worldwide.

## Supplementary Information

Below is the link to the electronic supplementary material.

Supplementary file1 (PDF 183 KB)

Supplementary file2 (PDF 257 KB)

Supplementary file3 (PDF 2258 KB)

Supplementary file4 (PDF 5588 KB)

## Data Availability

The study protocol was previously published on the Open Science Framework (OSF) and is available at DOI: (10.17605/OSF.IO/ENYGT). Other data supporting the findings of this study are available within the paper and its Supplementary Information.
