# Supplementary material for: Key performance indicators for hospital clinical pharmacy services: results of a global Delphi study
Source: Int J Clin Pharm. 2026 Apr 1;48(4):1489–99. doi: 10.1007/s11096-026-02126-y (PMC13368952; doi:10.1007/s11096-026-02126-y)
Supplement: Supplementary file 1 — Supplementary file1 (PDF 183 KB) [file 11096_2026_2126_MOESM1_ESM.pdf]

Supplementary file 1. Panellist recruitment (strategies and results)

Table 1 - Stakeholders groups and identification strategy (searches conducted in January 2024).

Stakeholder group	Panellist profile	Strategy of identification	Panellist identification
Clinical pharmacy experts	Professionals who participate in studies that address the development and implementation of clinical pharmacy KPIs in hospital settings	We employed the same strategy used in the review study: "Magedanz, L., Silva, H.L., Galato, D. et al. Clinical pharmacy key performance indicators for hospital inpatient setting: a systematic review. Int J Clin Pharm 46, 602–613 (2024). https://doi.org/10.1007/s11096-024-01717-x".	The names and e-mails of the authors of the selected studies were searched on the web.
Healthcare systems assessment experts	Professionals who participate in studies that discuss the evaluation of healthcare services in hospital settings	A search on Web Of Science was performed considering: "assessment" OR "evaluation" (Topic) AND "hospital" (Topic) AND "key performance indicators" OR "Performance" (Topic) AND "human resources" (Topic) and 2020 or 2021 or 2022 or 2023 (Publication Years)	The names and e-mails of the authors of the selected studies were searched on the web.
Patient Association's representatives	Patients and representatives of Patient Associations related to diseases that usually need hospital assistance or hospitalization	Using Google, a search on the web was performed considering the term "Patient association".	The organisations were contacted, by e-mail, to indicate representatives (only one representative per organisation).

Panellist selection process

After the initial verification, we found 480 potential candidates, distributed as follows: 116 clinical pharmacy experts; 179 healthcare systems assessment experts, and 185 patient associations.
An in-depth investigation allowed recovering 342 email contacts, of which 65 were clinical pharmacy experts, 118 healthcare systems assessment experts, and 159 patient associations.

- **Clinical pharmacy experts' studies:**
    - Ng J, Harrison J. Key performance indicators for clinical pharmacy services in New Zealand public hospitals: stakeholder perspectives. Journal of Pharmaceutical Health Services Research. 2010;1(2):75-84. doi: 10.1111/j.1759-8893.2010.00001.x
    - Doerper S, Morice S, Piney D, Dony A, Baum T, Perrin F, et al. La conciliation des traitements médicamenteux : logigramme d'une démarche efficiente pour prévenir ou intercepter les erreurs médicamenteuses à l'admission du patient hospitalisé. Le Pharmacien Hospitalier et Clinicien. 2013;48(3):153-60. doi: 10.1016/j.phclin.2013.03.006
    - Fernandes O, Gorman SK, Slavik RS, Semchuk WM, Shalansky S, Bussières JF, et al. Development of Clinical Pharmacy Key Performance Indicators for Hospital Pharmacists Using a Modified Delphi Approach. Canadian Journal of Hospital Pharmacy. 2016;69(1):55-. doi:
    - Aljamal MS, Ashcroft D, Tully MP. Development of indicators to assess the quality of medicines reconciliation at hospital admission: an e-Delphi study. Int J Pharm Pract. 2016;24(3):209-16. doi: 10.1111/ijpp.12234
    - Lloyd GF, Singh S, Barclay P, Goh S, Bajorek B. Hospital pharmacists' perspectives on the role of key performance indicators in Australian pharmacy practice. Journal of Pharmacy Practice and Research. 2016;47(2):87-95. doi: 10.1002/jppr.1156
    - Cillis M, Spinewine A, Krug B, Quennery S, Wouters D, Dalleur O. Development of a tool for benchmarking of clinical pharmacy activities. Int J Clin Pharm. 2018;40(6):1462-73. doi: 10.1007/s11096-018-0725-6
    - Krzyzaniak N, Pawlowska I, Bajorek B. Quality pharmacy services and key performance indicators in Polish NICUs: a Delphi approach. Int J Clin Pharm. 2018;40(3):533-42. doi: 10.1007/s11096-018-0623-y
    - Shawahna R. Development of Key Performance Indicators for Capturing Impact of Pharmaceutical Care in Palestinian Integrative Healthcare Facilities: A Delphi Consensus Study. Evid Based Complement Alternat Med. 2020;2020:7527543. doi: 10.1155/2020/7527543
    - Al-Jazairi AS, Alnakhli AO. Quantifying Clinical Pharmacist Activities in a Tertiary Care Hospital Using Key Performance Indicators. Hosp Pharm. 2021;56(4):321-7. doi: 10.1177/0018578719897074
    - King PK, Burkhardt C, Rafferty A, Wooster J, Walkerly A, Thurber K, et al. Quality measures of clinical pharmacy services during transitions of care. J American Coll Clin Pharm. 2021;4(7):883-907. doi: 10.1002/jac5.1479
    - Lopes H, Lopes AR, Farinha H, Martins AP. Defining clinical pharmacy and support activities indicators for hospital practice using a combined nominal and focus group technique. Int J Clin Pharm. 2021;43(6):1660-82. doi: 10.1007/s11096-021-01298-z

- Ruiz Ramos J, Calderon Hernanz B, Castellanos Clemente Y, Bonete Sanchez M, Vallve Alcon E, Santolaya Perrin Mf, et al. Pharmacist care in hospital emergency departments: a consensus paper from the Spanish hospital pharmacy and emergency medicine associations. Emergencias. 2023;35(3):205-17. doi:
- Anene-Okeke C. Clinical Pharmacy Key Performance Indicators (cpKPI) for Hospital Pharmacists in Nigeria. J Basic Clin Pharm. 2022;13(4):180-5. doi: 10.37532/0976-0113.13(4).180
- Canning, M.L., Barras, M., McDougall, R. et al. Defining quality indicators, pharmaceutical care bundles and outcomes of clinical pharmacy service delivery using a Delphi consensus approach. Int J Clin Pharm (2024). https://doi.org/10.1007/s11096-023-01681-y
- Acquisto NM, Beavers CJ, Bolesta S, et al. Development and application of quality measures of clinical pharmacist services provided in inpatient/acute care settings. J Am Coll Clin Pharm. 2021;4(12):1601-1617. doi:10.1002/jac5.1559

- **Healthcare systems assessment experts' studies:**

- Jafari, M; Seyedjavadi, M; Zaboli, R. Assessment of performance in teaching hospitals: Using multicriteria decision-making techniques. J EDUC HEALTH PROMOT. 2020. DOI: 10.4103/jehp.jehp_89_20. WOS:000570205600020
- Gaspar, T; Gomez-Baya, D; Guedes, FB; Correia, MF. Health Management: Evaluating the Relationship between Organizational Factors, Psychosocial Risks at Work, Performance Management, and Hospital Outcomes. HEALTHCARE-BASEL. 2023. DOI: 10.3390/healthcare11202744. WOS:001095210900001
- Meghdad, R; Nayereh, R; Zahra, S; Houriye, Z; Reza, N. Assessment of the performance of nurses based on the 360-degree model and fuzzy multi-criteria decision-making method (FMCDM) and selecting qualified nurses. HELIYON. 2020. DOI: 10.1016/j.heliyon.2020.e03257. WOS:000510380200210
- Aunión-Villa, J; Gómez-Chaparro, M; Sanz-Calcedo, JG. Assessment of the maintenance costs of electro-medical equipment in Spanish hospitals. EXPERT REV MED DEVIC. 2020. DOI: 10.1080/17434440.2020.1796635. WOS:000555619900001
- Yucesan, M; Gul, M; Celik, E. A multi-method patient arrival forecasting outline for hospital emergency departments. INT J HEALTHCARE MAN. 2020. DOI: 10.1080/20479700.2018.1531608. WOS:000607919000036
- Ta'an, WF; Al-Hammouri, MM; Al-Faouri, I; Suliman, MM. The effectiveness of COPA-based training program on the infection- control competencies of newly hired healthcare professionals. TEACH LEARN NURS. 2023. DOI: 10.1016/j.teln.2022.06.009. WOS:000931863600001
- Nuevo, M; Mahdavi, H; Rodríguez, D; Faura, T; Fabrellas, N; Balocco, S; Conti, M; Castagna, A; Prat, S. Evaluation of Safety and Efficacy of ReHub in Patients Who Underwent Primary Total Knee Arthroplasty: Study Protocol for a Randomized Controlled Trial. INT J SURG PROTOC. 2021. DOI: 10.29337/ijsp.138. WOS:000647604100006
- Okada, Y; Kiguchi, T; Irisawa, T; Yamada, T; Yoshiya, K; Park, C; Nishimura, T; Ishibe, T; Yagi, Y; Kishimoto, M; Inoue, T; Hayashi, Y; Sogabe, T; Morooka, T; Sakamoto, H; Suzuki, K; Nakamura, F; Matsuyama, T; Nishioka, N; Kobayashi, D; Matsui, S; Hirayama, A; Yoshimura, S; Kimata, S; Shimazu, T; Ohtsuru, S; Kitamura, T; Iwami, T. Development and Validation of a Clinical Score to Predict Neurological Outcomes in Patients With Out-of-Hospital Cardiac Arrest Treated With Extracorporeal

Cardiopulmonary Resuscitation. JAMA NETW OPEN. 2020. DOI: 10.1001/jamanetworkopen.2020.22920. WOS:000595939400003
- Lauck, SB; Thorne, SE; Saewyc, EM; Heppell, L; Black, A; Virani, SA. Promoting cardiovascular nursing practice and research: A model for a university joint appointment. J CLIN NURS. 2022. DOI: 10.1111/jocn.15588. WOS:000611688300001
- Annisa, F; Maidin, A; Mangilep, AUA; Irwandy. Performance evaluation of patient safety committee in the regional general hospital of Makassar city. ENFERM CLIN. 2020. DOI: 10.1016/j.enfcli.2020.06.003. WOS:000580629800002
- Taipa-Mendes, AM; Amaral, TF; Gregório, M. Undernutrition risk and nutritional screening implementation in hospitals: Barriers and time trends (2019-2020). CLIN NUTR ESPEN. 2021. DOI: 10.1016/j.clnesp.2021.08.029. WOS:000729956100022
- Letsio, A; Polyzos, N; Poulopoulos, C; Skamnakis, C. Hospital managers' participation in operational planning: insights from a recent study in the Greek National Health System. HIPPOKRATIA. 2022. DOI: . WOS:001023353200001
- Yakusheva, O; Bang, JT; Hughes, RG; Bobay, KL; Costa, L; Weiss, ME. Nonlinear association of nurse staffing and readmissions uncovered in machine learning analysis. HEALTH SERV RES. 2022. DOI: 10.1111/1475-6773.13695. WOS:000668447200001
- Mao, S; Soputhy, C; Lay, S; Jacobs, J; Ku, GM; Chau, D; Chhea, C; Ir, P. The barriers and facilitators of implementing a national laboratory-based AMR surveillance system in Cambodia: key informants' perspectives and assessments of microbiology laboratories. FRONT PUBLIC HEALTH. 2023. DOI: 10.3389/fpubh.2023.1332423. WOS:001135639900001
- Wenang, S; Schaefers, J; Afdal, A; Gufron, A; Geyer, S; Dewanto, I; Haier, J. Availability and Accessibility of Primary Care for the Remote, Rural, and Poor Population of Indonesia. FRONT PUBLIC HEALTH. 2021. DOI: 10.3389/fpubh.2021.721886. WOS:000704318500001
- Leon, N; Balakrishna, Y; Hohlfeld, A; Odendaal, WA; Schmidt, BM; Zweigenthal, V; Watkins, JA; Daniels, K. Routine Health Information System (RHIS) improvements for strengthened health system management. COCHRANE DB SYST REV. 2020. DOI: 10.1002/14551858.CD012012.pub2. WOS:000568684200001
- Wei, PL; Zhang, Y; Wu, SM; Wang, Q; Shui, GH; Su, CX; Han, EH; Dong, YX. Current Situation and Influencing Factors of Traditional Chinese Medicine Nursing Clinic in Henan Province. J HEALTHC ENG. 2022. DOI: 10.1155/2022/8941922. WOS:000802983700018
- Davies, JI; Reddiar, SK; Hirschhorn, LR; Ebert, C; Marcus, ME; Seiglie, JA; Zhumadilov, Z; Supiyev, A; Sturua, L; Silver, BK; Sibai, AM; Quesnel-Crooks, S; Norov, B; Mwangi, JK; Omar, OM; Wong-McClure, R; Mayige, MT; Martins, JS; Lunet, N; Labadarios, D; Karki, KB; Kagaruki, GB; Jorgensen, JMA; Hwalla, NC; Houinato, D; Houehanou, C; Guwatudde, D; Gurung, MS; Bovet, P; Bicaba, BW; Aryal, KK; Msaidié, M; Andall-Brereton, G; Brian, G; Stokes, A; Vollmer, S; Bärnighausen, T; Atun, R; Geldsetzer, P; Manne-Goehler, J; Jaacks, LM. Association between country preparedness indicators and quality clinical care for cardiovascular disease risk factors in 44 lower- and middle-income countries: A multicountry analysis of survey data. PLOS MED. 2020. DOI: 10.1371/journal.pmed.1003268. WOS:000590731500003
- Korouli, S; Kapaki, V; Egglezopoulou, A; Galanis, P. Implementing the Common Assessment Framework in a general hospital. ARCH HELL MED. 2020. DOI: . WOS:000538984500011

  - Vrabková, I; Vanková, I. Efficiency of Human Resources in Public Hospitals: An Example from the Czech Republic. INT J ENV RES PUB HE. 2021. DOI: 10.3390/ijerph18094711. WOS:000650238800001
  - Apornak, A; Raissi, S; Keramati, A; Khalili-Damghani, K. Optimizing human resource cost of an emergency hospital using multi-objective Bat algorithm. INT J HEALTHCARE MAN. 2021. DOI: 10.1080/20479700.2019.1707415. WOS:000505533200001
  - Yousefinezhadi, T; Mosadeghrad, AM; Hinchcliff, R; Akbari-Sari, A. Evaluation results of national hospital accreditation program in Iran: The view of hospital managers. J HEALTHC QUAL RES. 2020. DOI: 10.1016/j.jhqr.2019.08.008. WOS:000756537600003
  - Dada, A; Olaopa, OI; Chukwuanukwu, TO; Asumah, A. Building a Responsive Healthcare System - A Nigerian Experience. NIGER J CLIN PRACT. 2022. DOI: 10.4103/njcp.njcp_649_22. WOS:000923569200001
  - Lu, HQ; Wang, RL; Huang, ZJ. Application of Data Mining in Performance Management of Public Hospitals. MOB INF SYST. 2022. DOI: 10.1155/2022/2412928. WOS:000772198700002
  - Castanheira-Pinto, A; Gonçalves, BS; Lima, RM; Dinis-Carvalho, J. Modeling, Assessment and Design of an Emergency Department of a Public Hospital through Discrete-Event Simulation. APPL SCI-BASEL. 2021. DOI: 10.3390/app11020805. WOS:000610942600001

- **Patient Association's representatives:**
  - EL-AMEL Association
  - Linfomas Argentina
  - Australian patients association
  - Epilepsy Action Australia (IBE Associate Chapter)
  - Epilepsy Association of South Australia and the Northern Territory Inc.
  - Leukaemia Foundation of Australia
  - Lymphoma Australia
  - European Pulmonary Hypertension Association (PHA Europe)
  - Bangladesh Epilepsy Association
  - The Myeloma, Lymphoma and Leukaemia Foundation of Barbados
  - AGE Platform Europe (AGE)
  - Association of European Cancer Leagues (ECL)
  - European AIDS Treatment Group (EATG)
  - European Federation of Allergy and Airways Diseases Patients' Associations (EFA)
  - European Federation of Neurological Associations (EFNA)
  - European Haemophilia Consortium (EHC)
  - European Heart Network (EHN)
  - European Idiopathic Pulmonary Fibrosis & Related Disorders Federation (EU-IPFF)
  - European Liver Patient Association (ELPA)
  - European Lung Foundation (ELF)
  - European Multiple Sclerosis Platform (EMSP)
  - European Organisation for Rare Diseases (EURORDIS)
  - European Patients' Forum (EPF)
  - European Prostate Cancer Coalition (Europa Uomo)
  - European Public Health Alliance (EPHA)

- Global Alliance for Mental Illness Advocacy Networks (GAMIAN-Europe)
- Hodgkin en non-Hodgkin vzw
- International Diabetes Federation European Region (IDF Europe)
- International Patient Organisation for Primary Immunodeficiencies (IPOPI)
- International Prader-Willi Syndrome Organisation (IPWSO)
- Lymfklierkanker Vereniging Vlaanderen
- Myeloma Patients Europe (MPE)
- ABRALE Brazilian Lymphoma and Leukaemia Association
- Associação Brasileira de Epilepsia
- Bulgarian Lymphoma Patients' Association
- Children's Healthcare Canada Family Network
- CLL Canada
- GlobalSkin International Alliance of Dermatology Patient Organizations (IADPO)
- Lymphoma Canada
- Paediatric International Patient Safety and Quality Collaborative
- Patient Commando
- Patients Canada
- Patients for Patient Safety Canada
- The Center for Patient Protection
- Waldenstrom Macroglobulinemia Foundation of Canada (WMFC)
- Asociación Nacional de Ligas Chilenas contra la Epilepsia – ANLICHE
- China Association Against Epilepsy
- China Waldenstrom Macroglobulinemia Support Group
- Fundación Colombiana de Leucemia y Linfoma
- Fundación Instituto de Rehabilitación para Personas con Epilepsia – FIRE
- Asociación International Bureau for Epilepsy Capítulo Costa Rica
- Hrvatska Udruga Leukemija I Limfomi - Hull (Croatian Leukaemia and Lymphoma Society)
- Udruga Oboljelih od leukemije I limphoma (UOLL)/ Association of patients with leukemia and lymphoma
- Capitulo Cubano de la IBE
- Thalassaemia International Federation (TIF)
- Lymfom Help
- LyLe – Patientforeningen for Lymfekræft & Leukæmi
- Centro Nacional De Epilepsia – APNE
- Estonian Epilepsy Association
- Epilepsialiitto
- Suomen Syöpäpotilaat - Cancer patienterna i Finland (Association of Cancer Patients in Finland)
- ELLyE (Ensemble Leucémie, Lymphomes Espoir)
- Deutsche Leukäemie- & Lymphom-Hilfe eV
- European Foundation for the Care of Newborn Infants (EFCNI)
- European MEN Alliance (EMENA)
- Leukaemiehilfe RHEIN-MAIN e.V. (LHRM)
- CLL Greece (Hellenic Group of Patients with CLL)
- Hellenic Cancer Federation - ELLOK
- The Epilepsy Foundation of Guyana
- Cancer Patient Alliance

- Magyar Rákellenes Liga
- Lauf – The Icelandic Epilepsy Association
- Indian Epilepsy Association
- V Care Foundation
- Indonesian Cancer Information and Support Center Association (CISC for Lymphoma)
- International Bureau for Epilepsy (IBE)
- Lupus Europe
- Pain Alliance Europe (PAE)
- SMA Europe (SMAE)
- Leukemia Patient Care Organisation
- CLL Ireland
- European Institute of Women's Health (EIWH)
- Irish Cancer Society
- The Flute of Light
- Associazione Italiana contro le Leucemie-linfomi e mieloma – Pazienti (AIL)
- Gruppo Abruzzese Linfomi (GAL)
- Linfovita
- Jamaican Epilepsy Association
- Epilepsy Hospital Bethel (IBE Associate Chapter)
- Group Nexus Japan
- Japan Epilepsy Association Inc.
- Korea Blood Cancer Association
- Korea Bureau For Epilepsy (KBE)
- Limfomas pacientu atbalsta organizācija
- OHLB Kraujas
- Borka – for each new day
- Alzheimer Europe (AE)
- HEMA - Association for support of patients and their caregivers of Hematology diseases
- Majlis Kanser Nasional (MAKNA) (National Cancer Council)
- Asociacion Gerardo Alfaro A.C.
- Fundacion Nacional de Pacientes con Linfoma no Hodgkin (FunaLinH)
- Group “Acceptation” of Epilepsy (GADEP)
- Association ADAMS des maladies et du cancer du sang
- Ensemble Contre le Lymphome (ECL)
- European Huntington Association (EHA)
- Health Action International (HAI)
- Hematon
- Netherlands Patients Federation
- Patients Network for Medical Research and Health (EGAN)
- Stichting Huidlymfoom (The Dutch Cutaneous Lymphoma Foundation)
- Leukaemia & Blood Cancer New Zealand
- World Duchenne Organization (UPPMD)
- Ligue Nigérienne de Lutte contre le Cancer
- Fabry International Network (FIN)
- International Alliance of Patients' Organizations (IAPO)
- International Gaucher Alliance (IGA)
- Lung Cancer Europe (LuCE)

- Blodkreftforeningen
- Bureau Panameño contra la Epilepsia – BUPACE
- Asociación Paraguaya de Lucha contra la Epilepsia (ASOPALEP)
- Peruvian Association of Epilepsy
- Lymphoma Philippines
- Polish Lymphoma Association
- AAPC (Associação de Apoio a Pessoas com Cancro)
- ACREDITAR (Associação dos Pais e Amigos das Crianças com Cancro)
- ACRIM (Associação de Cancro do Rim de Portugal)
- Ame e viva a vida (Associação de mulheres mastectomizadas)
- ADL (Associação de Apoio aos Doentes com Leucemia e Linfoma)
- APAMCM (Associação Portuguesa de Apoio à Mulher com Cancro da Mama)
- APLL (Associação Portuguesa de Leucemias e Linfomas)
- Associacao Portuguesa Contra a Leucemia (APCL)
- Associação Portuguesa de Leucemias e Linfomas (APLL)
- Associação Portuguesa de ostomizados
- Europacolon Portugal (Apoio ao doente com cancro digestivo)
- FROC (Fundação Rui Osório de Castro)
- MyJourney – Associação Patient Advocacy
- LPCC (Liga Portuguesa Contra o Cancro)
- Pulmonale (Associação portuguesa de luta contra o cancro do pulmão)
- Lymphoma Patients Association of Romania
- Viva mulher viva (Associação para o bem-estar e qualidade de vida das mulheres com cancro da mama)
- Romanian Association against Leukemia
- All Russian Charity Foundation “Sodruzhestvo”
- Association of Doctors-Epileptologists and Patients
- Inter-regional Public Organization for Patients with Hematological Diseases Most Miloserdiya (Mercy Bridge)
- Inter-regional Public Organization for Patients with Hematological Diseases Most Miloserdiya (Mercy Bridge)
- LIPA Lymphoma Patient Association
- Association of Patients with Hematological Malignancies
- Leukemia & Lymphoma Foundation
- Lymfoma Slovensko
- Društvo bolnikov z limfomom
- Association of Patients with Blood Diseases Slovenia
- Slovensko združenje bolnikov z limfomom in levkemijo, L&L
- Campaigning for Cancer
- Epilepsy South Africa
- The Cancer Association of South Africa (CANSA)
- AEAL, Asociación Española de Afectados por Linfoma, Mieloma y Leucemia
- Spanish Network for Leukemia and Blood Disorders (AELCLES)
- Epilepsy Association of Sri Lanka
- Blodcancerförbundet
- Lymphome.ch Patientennetz Schweiz
- Formosa Cancer Foundation
- Blood Cancer Foundation of Tanzania (BCFT)

- Tunisian Epilepsy Association
- BIRKAN
- KanKo
- European Parkinson's Disease Association (EPDA)
- Leukaemia CARE
- Lymphoma Action
- The patients association
- Fundación Porsaleu
- CancerCare
- CLL Society
- Cutaneous Lymphoma Foundation
- Epilepsy Foundation
- International Waldenstrom’s Macroglobulinemia Foundation (IWMF)
- Lymphoma Research Foundation
- Patients Against Lymphoma (PAL)
- T-Cell Leukemia Lymphoma foundation
- The Leukemia & Lymphoma Society
- USA Patient Network
- World Patients Alliance
- Asociación Venezolana de Amigos con Linfoma
- Fundación Hemato-Oncológica Guyana (Fundahog)
- Venezuelan National Bureau
- Francisco Cruz Lima (Hosp. Albert Einstein - Brazil)
