# Supplementary material for: Key performance indicators for hospital clinical pharmacy services: results of a global Delphi study
Source: Int J Clin Pharm. 2026 Apr 1;48(4):1489–99. doi: 10.1007/s11096-026-02126-y (PMC13368952; doi:10.1007/s11096-026-02126-y)

Table 1. Geographical distribution of the participants of the Delphi survey.

Country	Participants who committed to participate in the Delphi panel (n = 49)	Participants who completed round 1 (n = 47)	Participants who completed round 2 (n = 43)	Participants who completed round 3 (n = 43)
Australia	5	5	4	4
Belgium	3	3	2	2
Brazil	1	1	1	1
Bulgaria	1	1	1	1
Canada	7	7	7	7
Colombia	1	1	1	1
Georgia	1	1	1	1
Greece	2	2	2	2
Guyana	1	1	1	1
Iran	2	2	2	2
Ireland	1	1	1	1
Mexico	1	1	1	1
Netherlands	1	1	1	1
New Zeland	1	1	1	1
Niger	1	1	0	0
Nigeria	1	1	1	1
Poland	1	1	1	1
Portugal	4	3	2	2
Russia	2	2	2	2
South Africa	1	1	1	1
Spain	5	5	5	5
Sri Lanka	1	1	1	1
Tunisia	1	1	1	1
United Kingdom	1	0	0	0
United States	3	3	3	3

Figure 1. Global distribution of panelists who completed the final round of the Delphi survey.

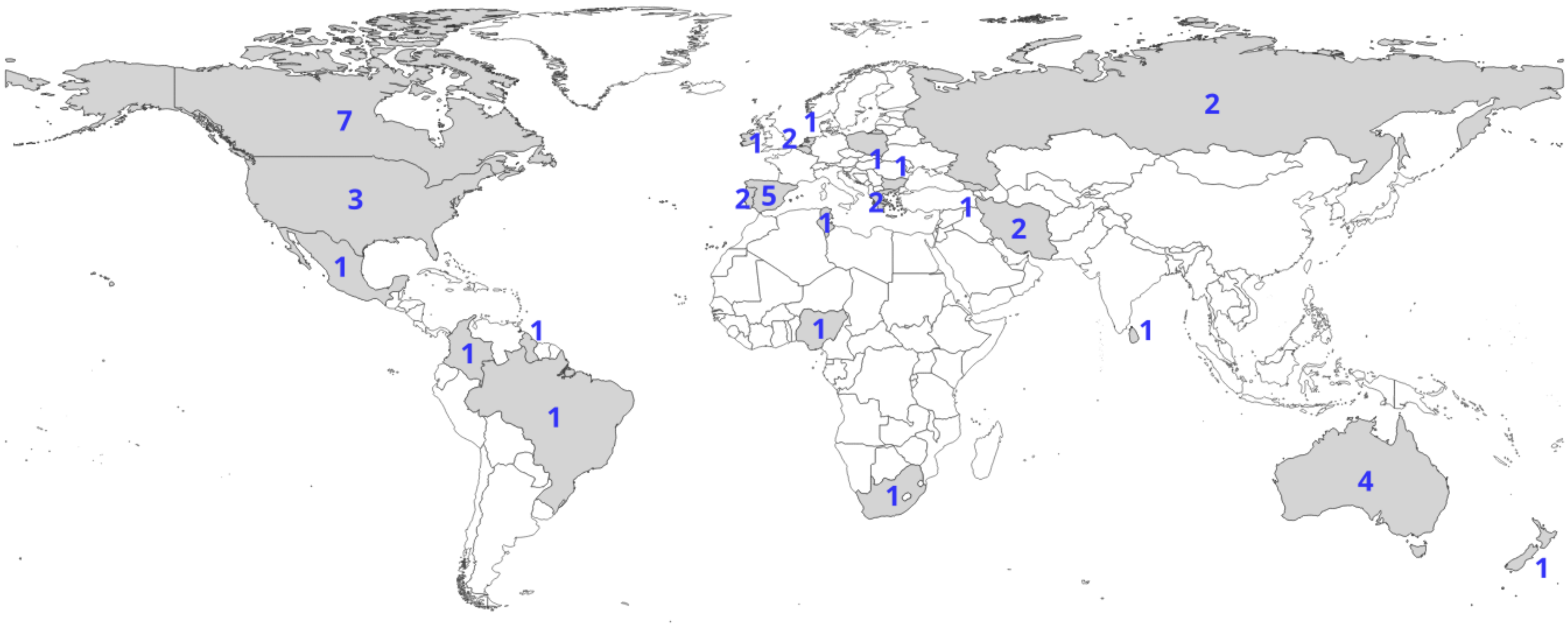

Supplement: Supplementary file 2 — Supplementary file2 (PDF 257 KB) [file 11096_2026_2126_MOESM2_ESM.pdf]
