# Supplementary material for: Key performance indicators for hospital clinical pharmacy services: results of a global Delphi study
Source: Int J Clin Pharm. 2026 Apr 1;48(4):1489–99. doi: 10.1007/s11096-026-02126-y (PMC13368952; doi:10.1007/s11096-026-02126-y)
Supplement: Supplementary file 3 — Supplementary file3 (PDF 2258 KB) [file 11096_2026_2126_MOESM3_ESM.pdf]

KPI C01

Round 1 KPIA proposal:

Section 3 - Clinic KPIs evaluation: KPI_C01

KPI: **Adverse drug event rate** (complete **information sheet** about this KPI is available here)

- Purpose: This KPI aims to evaluate the incidence rate of adverse drug events (ADEs) in patients followed by the hospital's Clinical Pharmacy Service (CPS).

$$\textbf{Adverse drug event rate} = \left\{ \frac{\text{number of adverse drug events (ADEs) that occurred in patients followed by the Clinical Pharmacy Service (CPS), in a certain period of time}}{\text{total number of patient days followed by the CPS in the hospital, in the same period of time}} \times 1000 \right\} = \textbf{Incidence rate of ADEs per 1,000 patient days followed by the CPS, in the hospital in a certain period of time}$$

Round 1 KPI relevance score: 72,3%

Round 1 KPI Improvements:
1. The KPI “information sheet” has been updated with more information in the "Operational considerations” section, including a new subsection of "KPI monitoring".

Round 2 KPI proposal: no changes (title, purpose, and calculation formula).

Round 2 KPI relevance score: 93,0%

Round 2 KPI Improvements:
1. The KPI “information sheet” has been updated with more information in the "KPI subvariations" encompassing the "Preventable adverse drug event rate" KPI subvariation.

Round 3 proposal: no changes (title, purpose, and calculation formula).

Round 3 KPI relevance score: 97,7%

KPI C02

Round 1 KPI proposal:

Section 3 - Clinic KPIs evaluation: KPI_C02

KPI: **Near miss events avoid rate** (complete **information sheet** about this KPI is available here)

- Purpose: This KPI aims to evaluate the near miss events avoid rate in patients followed by the hospital's Clinical Pharmacy Service (CPS).

$$\textbf{Near miss events avoid rate} = \left\{ \frac{\text{number of potential/suspected drug-related near-miss events verified in patients followed by the Clinical Pharmacy Service (CPS), in a certain period of time}}{\text{total number of patient days followed by the CPS in the hospital, in the same period of time}} \times 1000 \right\} = \textbf{Incidence rate of near miss event avoided per 1,000 patient-days followed by the CPS, in the hospital in a certain period of time}$$

Round 1 KPI relevance score: 68,1%

Round 1 KPI Improvements:

1. The name of the KPI was changed from "Near miss events avoid rate" to "Near miss event rate".
2. The term "potential" was removed from the numerator of the formula.
3. The term "verified' was replaced by “occurred" in the numerator of the formula.
4. The KPI “information sheet” has been updated with more information in the "Operational considerations” section, including a new subsection of "KPI monitoring".

Round 2 KPI proposal:

Section 1 - Clinic KPIs evaluation: KPI_C02

KPI: **Near miss event rate** (complete **KPI information sheet** about this KPI is available here)

- Purpose: This KPI aims to evaluate the near miss event rate that occurred in patients followed by the hospital's Clinical Pharmacy Service (CPS).

$$\textbf{Near miss event rate} = \left\{ \frac{\text{number of suspected drug-related near-miss events occurred in patients followed by the Clinical Pharmacy Service (CPS), in a certain period of time}}{\text{total number of patient days followed by the CPS in the hospital, in the same period of time}} \times 1000 \right\} = \textbf{Incidence rate of near miss event avoided per 1,000 patient-days followed by the CPS, in the hospital in a certain period of time}$$

Round 2 KPI relevance score: 76,7%

Round 2 KPI Improvements:

1. The name of the KPI was changed from "Near miss event rate" to "Medication-related near miss event rate".
2. The KPI “information sheet” has been updated concerning the "key concept".
3. The "examples" have been modified to more clinical pharmacy-sensitive situations.

Round 3 proposal:

Section 1 - Clinic KPIs evaluation: KPI_C02

KPI: **Medication-related near miss event rate** (complete **KPI information sheet** about this KPI is available here)

- Purpose: This KPI aims to evaluate medication-related near miss event rate that occurred in patients followed by the hospital's Clinical Pharmacy Service (CPS).

$$\textbf{Medication-related near miss event rate} = \left\{ \frac{\text{number of suspected medication-related near-miss events occurred in patients followed by the Clinical Pharmacy Service (CPS), in a certain period of time}}{\text{total number of patient days followed by the CPS in the hospital, in the same period of time}} \times 1000 \right\} = \textbf{Incidence rate of near miss event avoided per 1,000 patient-days followed by the CPS, in the hospital in a certain period of time}$$

Round 3 KPI relevance score: 93,0%

KPI C03

Round 1 KPI proposal:

Section 3 - Clinic KPIs evaluation: KPI_C03

KPI: **Length of stay** (complete **information sheet** about this KPI is available here)

- Purpose: This KPI aims to evaluate the average length of stay (in days) that patients followed by the hospital's Clinical Pharmacy Service (CPS) spent admitted to the hospital for treatment, care, or observation.

$$\text{Length of stay} = \left\{ \frac{\text{sum of the occupied hospital bed-days of the patients followed by the Clinical Pharmacy Service (CPS), in a certain period of time}}{\text{total number of patients admitted to the hospital and followed by the CPS, in the same period of time}} \right\} = \textbf{Average days of the length of stay of patients followed by the CPS, in the hospital in a certain period of time}$$

Round 1 KPI relevance score: 63,8%

Round 1 KPI Improvements:

1. The KPI “information sheet” has been updated with more information in the "Rational" and "Operational considerations” sections, including a new subsection of "KPI monitoring".

Round 2 KPI proposal: no changes (title, purpose, and calculation formula).

Round 2 KPI relevance score: 67,4%

Round 2 KPI Improvements:

1. The name of the KPI was changed from "Length of stay" to "Average length of stay".
2. The KPI “information sheet” has been updated with more information in the "KPI subvariations" encompassing a measure of saved days of length of stay.

Round 3 proposal: KPI did not participate in this Delphi round.

KPI C04

Round 1 KPI proposal:

Section 3 - Clinic KPIs evaluation: KPI_C04

KPI: **Drug therapeutic goal rate** (complete **information sheet** about this KPI is available here)

- Purpose: This KPI aims to evaluate the drug therapeutic goal rate in patients followed by the hospital's Clinical Pharmacy Service (CPS).

$$\text{Drug therapeutic goal rate} = \left\{ \frac{\text{number of drug therapies that achieved the planned therapeutic goal in patients followed by the Clinical Pharmacy Service (CPS), in a certain period of time}}{\text{total number of drug therapies followed by the CPS in the hospital, in the same period of time}} \times 100 \right\} = \text{Incidence rate of drug therapies that achieved the planned therapeutic goal per 100 therapies followed by the CPS, in the hospital in a certain period of time}$$

Round 1 KPI relevance score: 76,6%

Round 1 KPI Improvements:

1. The name of the KPI was changed from "Drug therapeutic goal rate" to "Drug therapeutic goal achievement rate".
2. The incidence rate was changed from “100” to “1,000”, standardizing the incidence rate across all Clinical Pharmacy KPIs.
3. The KPI “information sheet” has been updated with more information in the "Rational" and "Operational considerations” sections, including a new subsection of "KPI monitoring".

Round 2 KPI proposal:

Section 1 - Clinic KPIs evaluation: KPI_C04

KPI: **Drug therapeutic goal achievement rate** (complete **KPI information sheet** about this KPI is available here)

- Purpose: This KPI aims to evaluate the drug therapeutic goal achievement rate in patients followed by the hospital's Clinical Pharmacy Service (CPS).

$$\text{Drug therapeutic goal achievement rate} = \left\{ \frac{\text{number of drug therapies that achieved the planned therapeutic goal in patients followed by the Clinical Pharmacy Service (CPS), in a certain period of time}}{\text{total number of drug therapies followed by the CPS in the hospital, in the same period of time}} \times 1000 \right\} = \text{Incidence rate of drug therapies that achieved the planned therapeutic goal per 1,000 therapies followed by the CPS, in the hospital in a certain period of time}$$

Round 2 KPI relevance score: 83,7%

Round 2 KPI Improvements:

1. The name of the KPI was changed from "Drug therapeutic goal achievement rate" to "Medication goal achievement rate". This change has been implemented to standardize the term "medication" in substitution to "drug".

2. The KPI “information sheet” has been updated with more information about the key concept of "Medication goal", affirming that it encompasses objective/quantitative and subjective/qualitative planned goals.

Round 3 proposal:

Section 1 - Clinic KPIs evaluation: KPI_C04

KPI: **Medication goal achievement rate** (complete **KPI information sheet** about this KPI is available here)

- Purpose: This KPI aims to evaluate the medication goal achievement rate in patients followed by the hospital's Clinical Pharmacy Service (CPS).

$$\text{Medication goal achievement rate} = \left\{ \frac{\text{number of medications that achieved the planned goal in patients followed by the Clinical Pharmacy Service (CPS), in a certain period of time}}{\text{total number of drug therapies followed by the CPS in the hospital, in the same period of time}} \times 1000 \right\} = \text{Incidence rate of drug therapies that achieved the planned therapeutic goal per 1,000 therapies followed by the CPS, in the hospital in a certain period of time}$$

Round 3 KPI relevance score: 81,4%

KPI C05

Round 1 KPI proposal:

Section 3 - Clinic KPIs evaluation: KPI_C05

KPI: **Cancelled planned care due to suboptimal medicine management** (complete **information sheet** about this KPI is available here)

- Purpose: This KPI aims to evaluate the canceled planned care (e.g., canceled surgery, canceled discharge from ICU or hospital, etc.) due to suboptimal medicine management in patients followed by the hospital's Clinical Pharmacy Service (CPS).

$$\textbf{Cancelled planned care due to suboptimal medicine management} = \left\{ \frac{\text{number of canceled planned cares due to suboptimal medicine management in patients followed by the Clinical Pharmacy Service (CPS), in a certain period of time}}{\text{total number of patient days followed by the CPS in the hospital, in the same period of time}} \times 1000 \right\} = \textbf{Incidence rate of canceled planned care per 1,000 patient days followed by the CPS, in the hospital in a certain period of time}$$

Round 1 KPI relevance score: 44,7%

Round 1 KPI Improvements:

1. The name of the KPI was changed from "Cancelled planned care due to suboptimal medicine management" to "Medication-related cancellation of planned care rate".
2. The KPI “information sheet” has been updated with more information in the "Operational considerations” section, including a new subsection of "KPI monitoring".

Round 2 KPI proposal:

Section 1 - Clinic KPIs evaluation: KPI_C05

KPI: **Medication-related cancellation of planned care rate** (complete **KPI information sheet** about this KPI is available here)

- Purpose: This KPI aims to evaluate canceled planned care (e.g., canceled surgery, canceled discharge from ICU or hospital, etc.) due to suboptimal medicine management in patients followed by the hospital's Clinical Pharmacy Service (CPS).

$$\textbf{Medication-related cancellation of planned care rate} = \left\{ \frac{\text{number of canceled planned cares due to suboptimal medicine management in patients followed by the Clinical Pharmacy Service (CPS), in a certain period of time}}{\text{total number of patient days followed by the CPS in the hospital, in the same period of time}} \times 1000 \right\} = \textbf{Incidence rate of canceled planned care per 1,000 patient days followed by the CPS, in the hospital in a certain period of time}$$

Round 2 KPI relevance score: 62,8%

Round 2 KPI Improvements:

1. The name of the KPI was changed from "Medication-related cancellation of planned care rate" to "Medication-related canceled planned care rate".
2. The KPI “information sheet” has been updated, standardizing the term "medication" in substitution to "medicine".

Round 3 proposal: KPI did not participate in this Delphi round.

KPI C06

Round 1 KPI proposal:

Section 3 - Clinic KPIs evaluation: KPI_C06

KPI: **All-cause unplanned 30-day readmission rate** (complete **information sheet** about this KPI is available here)

- Purpose: This KPI aims to evaluate the rate of all-cause unplanned readmissions within 30 days, in a ward or emergency unit, in patients followed by the hospital's Clinical Pharmacy Service (CPS).

$$\text{All-cause unplanned 30-day readmission rate} = \left\{ \frac{\text{number of all-cause unplanned readmissions that occurred up to 30 days after hospital discharge in patients followed by the Clinical Pharmacy Service (CPS), in a certain period of time}}{\text{number of patients discharged from the hospital, in the same period of time}} \times 100 \right\} = \text{Incidence rate of all-cause unplanned 30-day readmissions per 100 patients followed by the CPS, in the hospital in a certain period of time}$$

Round 1 KPI relevance score: 68,1%

Round 1 KPI Improvements:

1. The current KPI numerator and "information sheet" have been delimited to only consider readmissions in hospital wards.
2. A new KPI was designed specifically to assess the "All-cause unplanned 30-day emergency department visit rate".
3. The incidence rate was changed from “100” to “1,000”, standardizing the incidence rate across all Clinical Pharmacy KPIs.
4. The KPI “information sheet” has been updated with more information in the "Operational considerations” section, including a new subsection of "KPI monitoring".

Round 2 KPI proposal:

Section 1 - Clinic KPIs evaluation: KPI_C06

KPI: **All-cause unplanned 30-day readmission rate** (complete **KPI information sheet** about this KPI is available here)

- Purpose: This KPI aims to evaluate the rate of all-cause unplanned readmissions in hospital wards within 30 days of a hospital discharge, in patients followed by the hospital's Clinical Pharmacy Service (CPS).

$$\text{All-cause unplanned 30-day readmission rate} = \left\{ \frac{\text{number of all-cause unplanned readmissions in hospital ward that occurred up to 30 days after hospital discharge in patients followed by the Clinical Pharmacy Service (CPS), in a certain period of time}}{\text{number of patients discharged from the hospital, in the same period of time}} \times 1000 \right\} = \text{Incidence rate of all-cause unplanned 30-day readmissions in hospital ward per 1,000 patients followed by the CPS, in the hospital in a certain period of time}$$

Round 2 KPI relevance score: 74,4%

Round 2 KPI Improvements:

1. The KPI “information sheet” has been updated with more subvariations, including an "All-cause unplanned 30-day readmission rate for the same reason that the last admission" (but

this metric requires a medical panel to judge each readmission to ensure that the new admission is related to the previous admission), and a "90-day readmission rate".

Round 3 proposal: no changes (title, purpose, and calculation formula).

Round 3 KPI relevance score: 74,4%

KPI C07

Round 1 KPI proposal:

Section 3 - Clinic KPIs evaluation: KPI_C07

KPI: **Mortality rate** (complete **information sheet** about this KPI is available here)

- Purpose: This KPI aims to evaluate the all-cause mortality rate in patients followed by the hospital's Clinical Pharmacy Service (CPS).

$$\textbf{Mortality rate} = \left\{ \frac{\text{number of deaths that occurred in patients followed by the Clinical Pharmacy Service (CPS), in a certain period of time]}}{\text{total number of patients admitted to the hospital and followed by the CPS, in the same period of time}} \times 100 \right\} = \textbf{Incidence rate of deaths per 100 admissions in patients followed by the CPS, in the hospital in a certain period of time}$$

Round 1 KPI relevance score: 63,8%

Round 1 KPI Improvements:

1. The name of the KPI was changed from "Mortality rate" to "All-cause mortality rate".
2. The incidence rate was changed from “100” to “1,000”, standardizing the incidence rate across all Clinical Pharmacy KPIs.
3. The KPI “information sheet” has been updated with more information in the "Operational considerations” section, including a new subsection of "KPI monitoring".

Round 2 KPI proposal:

KPI: **All-cause mortality rate** (complete **KPI information sheet** about this KPI is available here)

- Purpose: This KPI aims to evaluate the all-cause mortality rate in patients followed by the hospital's Clinical Pharmacy Service (CPS).

$$\textbf{All-cause mortality rate} = \left\{ \frac{\text{number of all-cause deaths that occurred in patients followed by the Clinical Pharmacy Service (CPS), in a certain period of time}}{\text{total number of patients admitted to the hospital and followed by the CPS, in the same period of time}} \times 1000 \right\} = \textbf{Incidence rate of deaths per 1,000 admissions in patients followed by the CPS, in the hospital in a certain period of time}$$

Round 2 KPI relevance score: 79,1%

Round 2 KPI Improvements: No improvements/changes have been implemented in this round.

Round 3 proposal: no changes (title, purpose, and calculation formula).

Round 3 KPI relevance score: 76,7%

KPI C08

Round 1 KPI proposal: KPI did not participate in this Delphi round.

Round 2 KPI proposal:

KPI: **All-cause unplanned 30-day emergency department visit rate** (complete **KPI information sheet** about this KPI is available here)

- Purpose: This KPI aims to evaluate the rate of all-cause unplanned visits in emergency departments of hospital within 30 days of a hospital discharge, in patients followed by the hospital's Clinical Pharmacy Service (CPS).

$$\textbf{All-cause unplanned 30-day emergency department visit rate} = \left\{ \frac{\text{number of all-cause unplanned emergency department visit that occurred up to 30 days after hospital discharge in patients followed by the Clinical Pharmacy Service (CPS), in a certain period of time}}{\text{number of patients discharged from the hospital, in the same period of time}} \times 1000 \right\} = \textbf{Incidence rate of all-cause unplanned 30-day emergency department visit per 1,000 patients followed by the CPS, in the hospital in a certain period of time}$$

Round 2 KPI relevance score: 76,7%

Round 2 KPI Improvements:

1. The name of the KPI was changed from "All-cause unplanned 30-day emergency department visit rate" to "All-cause 30-day emergency department visit rate", considering that an emergency visit is always unplanned.
2. The KPI “information sheet” has been updated with more subvariations, including an "All-cause unplanned 30-day readmission rate for the same reason that the last admission" (but this metric requires a medical panel to judge each readmission to ensure that the new admission is related to the previous admission), and a "90-day readmission rate".

Round 3 proposal:

KPI: **All-cause 30-day emergency department visit rate** (complete **KPI information sheet** about this KPI is available here)

- Purpose: This KPI aims to evaluate the rate of all-cause visits in emergency departments of hospitals within 30 days of a hospital discharge, in patients followed by the hospital's Clinical Pharmacy Service (CPS).

$$\textbf{All-cause 30-day emergency department visit rate} = \left\{ \frac{\text{number of all-cause emergency department visit that occurred up to 30 days after hospital discharge in patients followed by the Clinical Pharmacy Service (CPS), in a certain period of time}}{\text{number of patients discharged from the hospital, in the same period of time}} \times 1000 \right\} = \textbf{Incidence rate of all-cause unplanned 30-day emergency department visit per 1,000 patients followed by the CPS, in the hospital in a certain period of time}$$

Round 3 KPI relevance score: 72,1%

KPI E01

Round 1 KPI proposal:

Section 4 - Economic KPIs evaluation: KPI_E01

KPI: **Billing/invoice generated** (complete **information sheet** about this KPI is available here)

- Purpose: This KPI aims to calculate the billing/invoice generated by the hospital's Clinical Pharmacy Service (CPS).

$$\text{Billing/invoice generated} = \left\{ \text{Sum of amounts billed/invoiced by the Clinical Pharmacy Service (CPS) in the hospital, in a certain period of time} \right\} = \textbf{Total billing generated (in US dollars or local currency) generated by the CPS, in the hospital in a certain period of time}$$

Round 1 KPI relevance score: 46,8%

Round 1 KPI Improvements:
1. The name of the KPI was changed from "Billing/invoice generated" to "Clinical Pharmacy Service revenue generated".
2. The KPI was "standardized" to an incidence rate of 1,000 patient days followed by the Clinical Pharmacy Service (CPS).
3. The KPI "information sheet" has been updated with more information in the "Operational considerations" section, including a new subsection of "KPI monitoring".

Round 2 KPI proposal:

Section 2 - Economic KPIs evaluation: KPI_E01

KPI: **Clinical Pharmacy Service revenue generated** (complete **KPI information sheet** about this KPI is available here)

- Purpose: This KPI aims to calculate the revenue generated by the hospital's Clinical Pharmacy Service (CPS).

$$\text{Clinical Pharmacy Service revenue generated} = \left\{ \frac{\text{Sum of amounts billed/invoiced by the Clinical Pharmacy Service (CPS) in the hospital, in a certain period of time}}{\text{total number of patient days followed by the CPS in the hospital, in the same period of time}} \times 1000 \right\} = \textbf{Estimated revenue generated (in US dollars or local currency) per 1,000 patient days followed by the CPS, in the hospital in a certain period of time}$$

Round 2 KPI relevance score: 55,8%

Round 2 KPI Improvements:
1. The name of the KPI was changed from "Clinical Pharmacy Service revenue generated" to "Average Clinical Pharmacy Service revenue generated".

Round 3 proposal: KPI did not participate in this Delphi round.

KPI E02

Round 1 KPI proposal:

Section 4 - Economic KPIs evaluation: KPI_E02

KPI: **Total optimized treatment cost savings** (complete **information sheet** about this KPI is available here)

- Purpose: This KPI aims to calculate the difference in cost between a given medication ("original drug therapy") and an "alternative treatment" (including changes in drug, dose, regime, etc.) suggested by the hospital's Clinical Pharmacy Service (CPS) and accepted by the healthcare team.

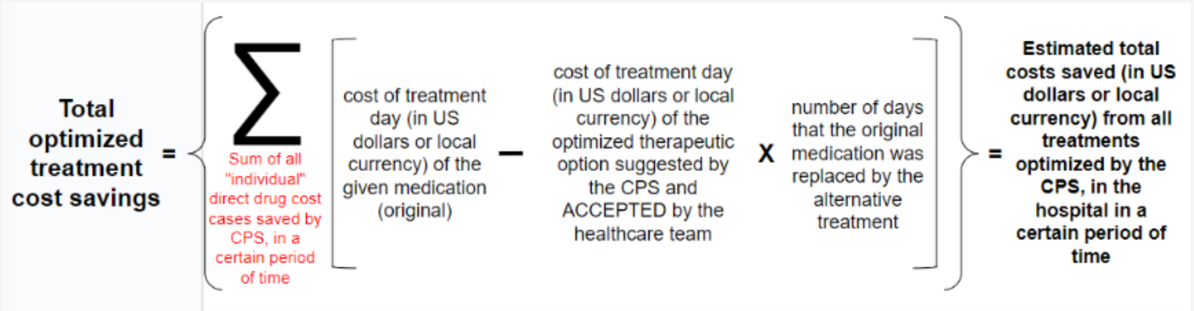

Round 1 KPI relevance score: 76,6%

Round 1 KPI Improvements:

1. The KPI formula has been corrected by adding parentheses between the numerator terms.
2. The KPI “information sheet” has been updated with more information in the "Operational considerations” section, including a new subsection of "KPI monitoring".

Round 2 KPI proposal:

Section 2 - Economic KPIs evaluation: KPI_E02

KPI: **Total optimized treatment cost savings** (complete **KPI information sheet** about this KPI is available here)

- Purpose: This KPI aims to calculate the difference in cost between a given medication ("original drug therapy") and an "alternative treatment" (including changes in drug, dose, regime, etc.) suggested by the hospital's Clinical Pharmacy Service (CPS) and accepted by the healthcare team.

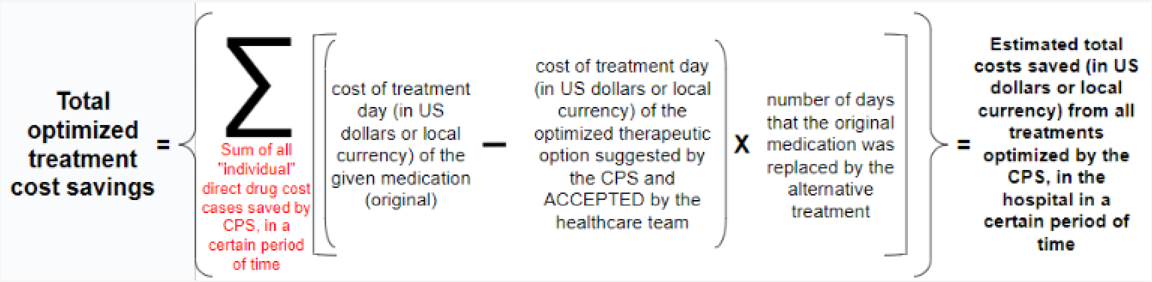

Round 2 KPI relevance score: 81,4%

Round 2 KPI Improvements:

1. The name of the KPI was changed from "Total optimized treatment cost savings" to "Total optimized treatment cost difference".
2. The KPI formula has been updated, changing the order of the terms to make the interpretation of the results easy. A "positive" result means that the optimized treatment had a greater financial cost compared to the original treatment. A "negative" result demonstrates direct cost savings.

3. The KPI “information sheet” has been updated with a "KPI interpretation" section.

Round 3 proposal:

Section 2 - Economic KPIs evaluation: KPI_E02

KPI: **Total optimized treatment cost difference** (complete **KPI information sheet** about this KPI is available here)

- Purpose: This KPI aims to calculate the difference in cost between a given medication ("original medication") and an "optimized treatment" (including changes in drug, dose, regime, and even discontinuation of the medication) suggested by the hospital's Clinical Pharmacy Service (CPS) and accepted by the healthcare team.

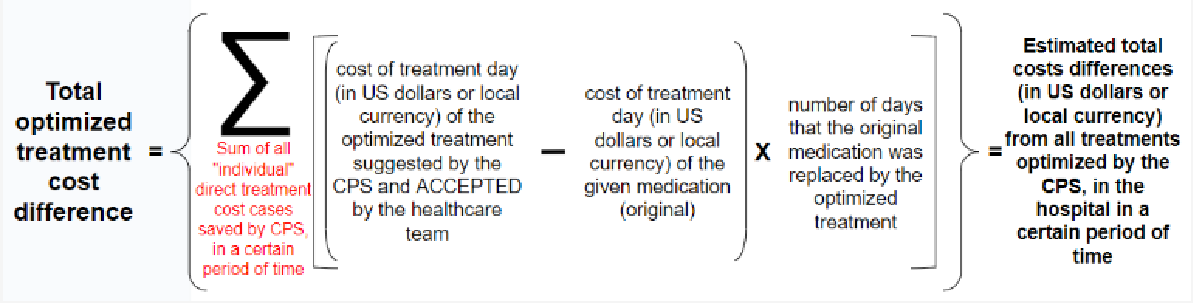

Round 3 KPI relevance score: 74,4%

KPI E03

Round 1 KPI proposal:

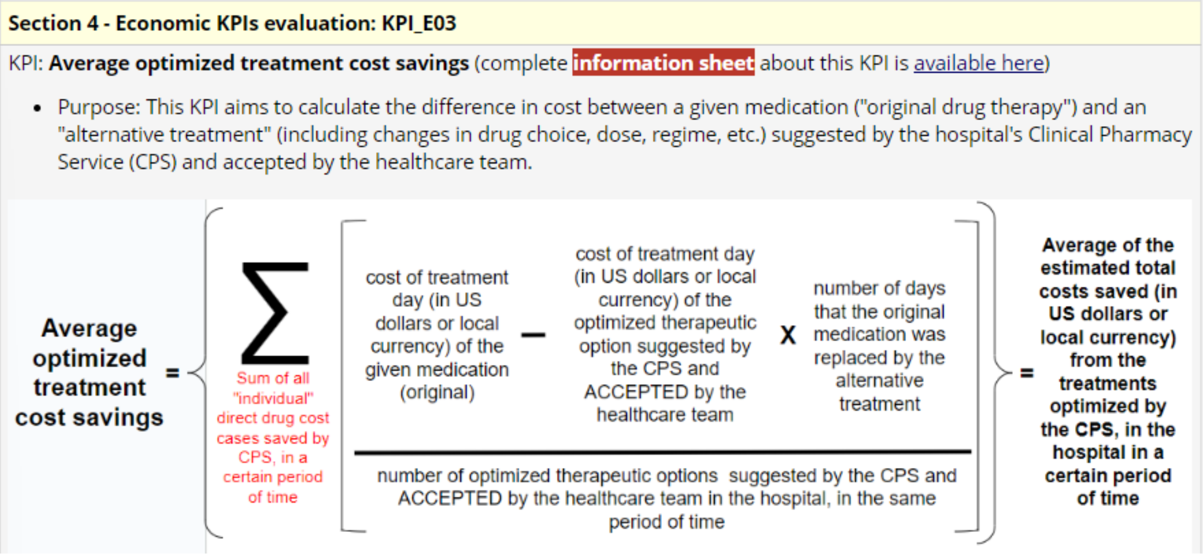

Round 1 KPI relevance score: 68,1%

Round 1 KPI Improvements:

1. The KPI formula has been corrected by adding parentheses between the numerator terms.
2. The KPI “information sheet” has been updated with more information in the "Operational considerations” section, including a new subsection of "KPI monitoring".

Round 2 KPI proposal:

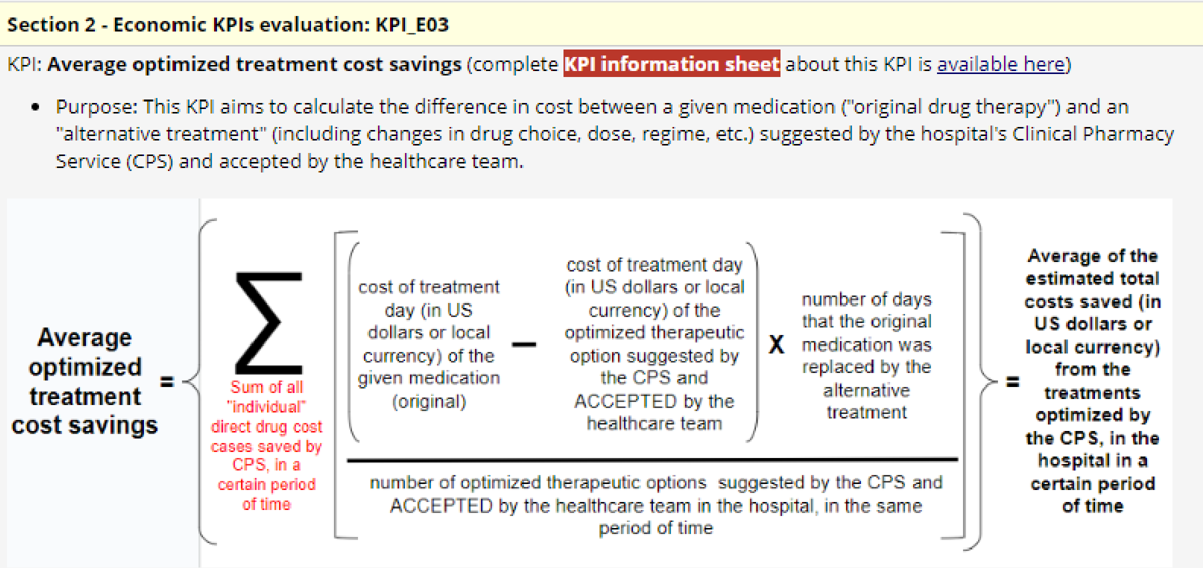

Round 2 KPI relevance score: 81,4%

Round 2 KPI Improvements:

1. The name of the KPI was changed from "Average optimized treatment cost savings" to "Average optimized treatment cost difference".

2. The KPI formula has been updated, changing the order of the therms to make easy the interpretation of the results. A "positive" result means that the optimized treatment had a greater financial cost compared to the original treatment. A "negative" result demonstrates direct cost savings.
3. The KPI “information sheet” has been updated with a "KPI interpretation" section.
4. A "Median optimized treatment difference" was suggested in KPI subvariations.

Round 3 proposal:

Section 2 - Economic KPIs evaluation: KPI_E03

KPI: **Average optimized treatment cost difference** (complete **KPI information sheet** about this KPI is available here)

- Purpose: This KPI aims to calculate the difference in cost between a given medication ("original medication") and an "optimized treatment" (including changes in drug, dose, regime, and even discontinuation of the medication) suggested by the hospital's Clinical Pharmacy Service (CPS) and accepted by the healthcare team.

$$\textbf{Average optimized treatment cost difference} = \left\{ \sum_{\substack{\text{Sum of all "individual" direct drug cost}\\ \text{cases saved by CPS, in a certain period of time}}} \left[\frac{\left(\begin{array}{c}\text{cost of treatment day (in US dollars or local currency) of the}\\ \text{optimized treatment suggested by the CPS and ACCEPTED by the healthcare team}\end{array} - \begin{array}{c}\text{cost of treatment day (in US dollars or local}\\ \text{currency) of the given medication (original)}\end{array} \right) \times \begin{array}{c}\text{number of days that the original medication}\\ \text{was replaced by the optimized treatment}\end{array}}{\text{number of optimized therapeutic options suggested by the CPS and ACCEPTED by the healthcare team in the hospital, in the same period of time}} \right] \right\} = \textbf{Average of the estimated total costs differences (in US dollars or local currency) from the treatments optimized by the CPS, in the hospital in a certain period of time}$$

Round 3 KPI relevance score: 81,4%

KPI H01

Round 1 KPI proposal:

Section 5 - Humanistic KPIs evaluation: KPI_H01

KPI: **Patient Reported Experience Measures (PREMs)** (complete **information sheet** about this KPI is available here)

- Purpose: This KPI aims to evaluate the frequency of complaints and Patient Reported Experience Measures (PREMs) related to Clinical Pharmacy Services (CPSs) in patients followed by the hospital's CPS.

$$\text{Patient reported experience measures (PREMs)} = \left\{ \begin{array}{c} \text{Total number of complaints and NEGATIVE Patient Reported Experience} \\ \text{Measures (PREMs) related to the Clinical Pharmacy Service (CPS) in the hospital,} \\ \text{in a certain period of time} \end{array} \right\} = \text{Total number of complaints and negative PREMs related to the CPS in the hospital, in a certain period of time}$$

Round 1 KPI relevance score: 74,5%

Round 1 KPI Improvements:

1. The KPI formula has been updated to consider the incidence of negative PREMs in relation to total PREMs (positive + negative).
2. The term "complains" has been removed from the numerator, as it is intrinsic to “negative PREMs”.
3. The KPI was "standardized" to an incidence rate of NEGATIVE PREMs per 1,000 PREMs related to the Clinical Pharmacy Service (CPS).
4. The KPI “information sheet” has been updated with more information in the "Operational considerations” section, including a new subsection of "KPI monitoring".

Round 2 KPI proposal:

Section 3 - Humanistic KPIs evaluation: KPI_H01

KPI: **Negative Patient Reported Experience Measures (PREMs) rate** (complete **KPI information sheet** about this KPI is available here)

- Purpose: This KPI aims to evaluate the frequency of negative Patient Reported Experience Measures (PREMs) related to Clinical Pharmacy Service (CPS).

$$\text{Negative Patient Reported Experience Measures (PREMs) rate} = \left\{ \frac{\text{Total number NEGATIVE Patient Reported Experience Measures (PREMs) related to the Clinical Pharmacy Service (CPS) in the hospital, in a certain period of time}}{\text{Total number of PREMs (positive + negative) related to the CPS in the hospital, in the same period of time}} \times 1000 \right\} = \text{Incidence rate of NEGATIVE PREMs per 1,000 PREMs related to the CPS, in the hospital, in a certain period of time}$$

Round 2 KPI relevance score: 76,7%

Round 2 KPI Improvements:

1. The KPI “information sheet” has been updated with more information in the "Operational considerations” section, including considerations about the Likert scale applied to PREMs.

Round 3 proposal: no changes (title, purpose, and calculation formula).

Round 3 KPI relevance score: 74,4%

KPI E02

Round 1 KPI proposal:

Section 5 - Humanistic KPIs evaluation: KPI_H02

KPI: **Patient satisfaction** (complete **information sheet** about this KPI is available here)

- Purpose: This KPI aims to evaluate the satisfaction of the patients followed by the hospital's Clinical Pharmacy Service (CPS).

Patient satisfaction = { Results of satisfaction surveys collected from patients followed by the Clinical Pharmacy Service (CPS) in the hospital, in a certain period of time } = **Satisfaction perception in patients followed by the CPS in the hospital, in a certain period of time**

Round 1 KPI relevance score: 72,3%

Round 1 KPI Improvements:

1. The name of the KPI was changed from "Patient satisfaction" to "Patient satisfaction surveys results".
2. The KPI "information sheet" has been updated with more information in the "Operational considerations" section, including a new subsection of "KPI monitoring".

Round 2 KPI proposal:

Section 3 - Humanistic KPIs evaluation: KPI_H02

KPI: **Patient satisfaction survey results** (complete **KPI information sheet** about this KPI is available here)

- Purpose: This KPI aims to evaluate the results of satisfaction surveys applied to patients followed by the hospital's Clinical Pharmacy Service (CPS).

Patient satisfaction surveys results = { Results of satisfaction surveys collected from patients followed by the Clinical Pharmacy Service (CPS) in the hospital, in a certain period of time } = **Satisfaction perception in patients followed by the CPS in the hospital, in a certain period of time**

Round 2 KPI relevance score: 86,0%

Round 2 KPI Improvements:

1. The KPI "information sheet" has been updated with more information in the "Operational considerations" section, including considerations about the Likert scale applied to patient's satisfaction evaluations.

Round 3 proposal: no changes (title, purpose, and calculation formula).

Round 3 KPI relevance score: 93,0%
