# Supplementary material for: Key performance indicators for hospital clinical pharmacy services: results of a global Delphi study
Source: Int J Clin Pharm. 2026 Apr 1;48(4):1489–99. doi: 10.1007/s11096-026-02126-y (PMC13368952; doi:10.1007/s11096-026-02126-y)
Supplement: Supplementary file 4 — Supplementary file4 (PDF 5588 KB) [file 11096_2026_2126_MOESM4_ESM.pdf]

KPI information sheet

Clinical Pharmacy KPI: **Adverse drug event rate**

Version: 03	Date: April / 2024	Page 1/2

Purpose: This KPI aims to evaluate the incidence rate of adverse drug events (ADEs) in patients followed by the hospital's Clinical Pharmacy Service (CPS).

Calculation formula:

$$\text{Adverse drug event rate} = \left\{ \frac{\text{number of adverse drug events (ADEs) that occurred in patients followed by the Clinical Pharmacy Service (CPS), in a certain period of time}}{\text{total number of patient days followed by the CPS in the hospital, in the same period of time}} \times 1000 \right\} = \text{Incidence rate of ADEs per 1,000 patient days followed by the CPS, in the hospital in a certain period of time}$$

Key concepts:

- **Adverse drug event (ADE):** any harmful or unintended medical occurrence related to the use of medication, including prescription drugs, over-the-counter medications, herbal remedies, and dietary supplements. ADEs can encompass a wide range of negative outcomes, including adverse drug reactions, medication errors, allergic reactions, falls, and other undesirable effects.

Rational:

- This KPI provides valuable insights into the level of medication safety practices in the hospital. By monitoring ADEs as a KPI, hospitals can enhance patient safety, optimize medication management processes, and improve overall healthcare quality and outcomes.
- ADEs are always understood as a negative outcome, and their avoidance is one of the main objectives of pharmaceutical care.

Operational considerations:

- **Category of the indicator:** clinic outcome.
- **Frequency of measure:** monthly.
- **Polarity of the measure:** the less, the better.
    - Important note: It is crucial to note that while the polarity suggests a logical "the less, the better" approach, the evaluation of data should be conducted with caution. Initially, in the first months of CPS, the KPI may increase due to improved and structured recording of ADEs and the growing expertise of clinical pharmacists in identifying ADEs. However, over a longer evaluation period, the validity of the polarity strengthens as CPS demonstrates its ability to prevent ADEs.

CONFIDENTIAL

The Clinical Pharmacy KPI research team

Msc. Pharm. Lucas Magedanz (https://orcid.org/0000-0001-9812-2783)
Dr.ª Pharm. Dayani Galato (https://orcid.org/0000-0002-9295-8018)
Dr. Fernando Fernandez-Llimos (https://orcid.org/0000-0002-8529-9595)

CONFIDENTIALITY NOTICE
This document contains confidential information intended solely for the recipient and may be legally privileged

- **KPI monitoring:** it is expected that the monthly results measured by the KPI will be compared with each other (longitudinal monitoring).
  - An alternative approach considers evaluating the same indicator in “patients not followed by the CPS”, thus creating a cohort comparator (transversal monitoring). This approach, however, must be carefully evaluated, especially when the epidemiological characteristics of the two groups (CPS-followed and non-CPS-followed patients) are different.
- **KPI subvariations:** subvariations can be created by considering risk adjustments due to the hospital's epidemiology and specialty (see examples below). However, the use of KPI subvariations depends on an adequate definition of the limiting concepts introduced both in the numerator and denominator. Also, they should be interpreted as “complementary” to the overall assessment of the Clinical Pharmacy Service that is measured in the main KPI.
  - Examples:
    - Adverse drug event rate in patients admitted to Intensive Care Units (ICUs) *(specific ward risk adjustment)*
    - Adverse drug event rate in pediatric patients *(specific age risk adjustment)*
    - Adverse drug event rate in oncologic patients *(specific disease risk adjustment)*
    - Critical and severe harm adverse drug event rate *(specific type of ADE risk adjustment)*
      - The concept of “critical” and “severe” adverse drug events must be defined in advance.
    - Preventable adverse drug event rate *(specific type of ADE risk adjustment)*
      - The concept of “preventable” must be defined in advance and may vary between hospitals due to technological resources.

CONFIDENTIAL

The Clinical Pharmacy KPI research team

Msc. Pharm. Lucas Magedanz (https://orcid.org/0000-0001-9812-2783)
Dr.ª Pharm. Dayani Galato (https://orcid.org/0000-0002-9295-8018)
Dr. Fernando Fernandez-Llimos (https://orcid.org/0000-0002-8529-9595)

CONFIDENTIALITY NOTICE
This document contains confidential information intended solely for the recipient and may be legally privileged

**KPI information sheet**		
Clinical Pharmacy KPI: **Medication-related near miss event rate**		
Version: 03	Date: April / 2024	

Purpose: This KPI aims to evaluate medication-related near miss event rate that occurred in patients followed by the hospital's Clinical Pharmacy Service (CPS).

Calculation formula:

$$\textbf{Medication-related near miss event rate} = \left\{ \frac{\text{number of suspected medication-related near-miss events occurred in patients followed by the Clinical Pharmacy Service (CPS), in a certain period of time}}{\text{total number of patient days followed by the CPS in the hospital, in the same period of time}} \times 1000 \right\} = \textbf{Incidence rate of near miss event avoided per 1,000 patient-days followed by the CPS, in the hospital in a certain period of time}$$

Key concepts:

- **Medication-related near miss events:** situations where there is a potential for harm to a patient or compromise patient safety due to a medication error or mistake, but were intercepted or mitigated before reaching the patient. Examples of near miss events include:
    - Medication Errors: the physician prescribes a dose of medication to the patient, but the pharmacist finds that the dose is not corrected for kidney function. After contacting the physician, the dose is corrected in the prescription before being administered.
    - Communication Breakdowns: the physician orders medication for a patient, but the pharmacist notices a discrepancy between the medication order and the patient's medical history. The pharmacist contacts the physician to clarify the order, preventing the patient from receiving the wrong medication.
    - Communication Breakdowns: the pharmacist checks and corrects the patient's documented home medication list (admission reconciliation), but the physician does not update the dose/frequency of prescribed medications (assuming the home medication list was correct), but the pharmacist notes this discrepancy and contact the doctor to correct the prescription before administering the medicine.

Rational:

- This KPI helps healthcare organizations identify gaps, vulnerabilities, and areas for improvement in patient care processes, systems, and workflows. By tracking this

The Clinical Pharmacy KPI research team

Msc. Pharm. Lucas Magedanz (https://orcid.org/0000-0001-9812-2783)
Dr.ª Pharm. Dayani Galato (https://orcid.org/0000-0002-9295-8018)
Dr. Fernando Fernandez-Llimos (https://orcid.org/0000-0002-8529-9595)

CONFIDENTIALITY NOTICE
This document contains confidential information intended solely for the recipient and may be legally privileged

KPI, organizations can implement targeted interventions and risk mitigation strategies to reduce the likelihood of near miss events occurring in the future.

Operational considerations:

- **Category of the indicator:** clinic outcome.
- **Frequency of measure:** monthly.
- **Polarity of the measure:** the less, the better.
    - Important note: It is crucial to note that while the polarity suggests a logical "the less, the better" approach, the evaluation of data should be conducted with caution. Initially, in the first months of CPS, the KPI may increase due to improved and structured recording of Near miss events and the growing expertise of clinical pharmacists in identifying them. However, over a longer evaluation period, the validity of the polarity strengthens as CPS demonstrates its ability to prevent Near miss events.
- **KPI monitoring: it** is expected that the monthly results measured by the KPI will be compared with each other (longitudinal monitoring).
    - An alternative approach considers evaluating the same indicator in “patients not followed by the CPS”, thus creating a cohort comparator (transversal monitoring). This approach, however, must be carefully evaluated, especially when the epidemiological characteristics of the two groups (CPS-followed and non-CPS-followed patients) are different.
- **KPI subvariations:** subvariations can be created by considering risk adjustments due to the hospital's epidemiology and specialty (see examples below). However, the use of KPI subvariations depends on an adequate definition of the limiting concepts introduced both in the numerator and denominator. Also, they should be interpreted as “complementary” to the overall assessment of the Clinical Pharmacy Service.
    - Examples:
        - Near miss event rate in patients admitted to Intensive Care Units (ICUs) *(specific ward risk adjustment)*
        - Near miss event rate in pediatric patients *(specific age risk adjustment)*
        - Near miss event rate in oncologic patients *(specific disease risk adjustment)*

The Clinical Pharmacy KPI research team

Msc. Pharm. Lucas Magedanz (https://orcid.org/0000-0001-9812-2783)
Dr.ª Pharm. Dayani Galato (https://orcid.org/0000-0002-9295-8018)
Dr. Fernando Fernandez-Llimos (https://orcid.org/0000-0002-8529-9595)

CONFIDENTIALITY NOTICE
This document contains confidential information intended solely for the recipient and may be legally privileged

CONFIDENTIAL

Purpose: This KPI aims to evaluate the average length of stay (in days) that patients followed by the hospital's Clinical Pharmacy Service (CPS) spent admitted to the hospital for treatment, care, or observation.

Calculation formula:

$$\textbf{Average length of stay} = \left\{ \frac{\text{sum of the occupied hospital bed-days of the patients followed by the Clinical Pharmacy Service (CPS), in a certain period of time}}{\text{total number of patients admitted to the hospital and followed by the CPS, in the same period of time}} \right\} = \textbf{Average days of the length of stay of patients followed by the CPS, in the hospital in a certain period of time}$$

Key concepts:

- **Length of stay (LOS):** the duration of time that a patient spends admitted to a hospital or healthcare facility for treatment, care, or observation.

Rational:

- This KPI provides insight into the efficiency and effectiveness of care delivery processes within the hospital. A shorter length of stay indicates that patients are receiving timely and appropriate care, leading to quicker recovery and discharge, which ultimately enhances overall efficiency in resource utilization and patient throughput. By tracking length of stay as a KPI, hospitals can optimize resource allocation, reduce unnecessary costs associated with prolonged hospitalizations, and ensure efficient use of available resources to meet patient needs.

Operational considerations:

- **Category of the indicator:** clinic outcome.
- **Frequency of measure:** monthly.
- **Polarity of the measure:** the less, the better.
  - Important note: Although we recognize that CPS is not the only preponderant factor in LOS, studies have shown that CPS can modify this variable.
- **KPI monitoring:** it is expected that the monthly results measured by the KPI will be compared with each other (longitudinal monitoring).
  - An alternative approach considers evaluating the same indicator in “patients not followed by the CPS”, thus creating a cohort comparator (transversal monitoring). This approach, however, must be carefully evaluated, especially when the

CONFIDEN

The Clinical Pharmacy KPI research team

Msc. Pharm. Lucas Magedanz (https://orcid.org/0000-0001-9812-2783)
Dr.ª Pharm. Dayani Galato (https://orcid.org/0000-0002-9295-8018)
Dr. Fernando Fernandez-Llimos (https://orcid.org/0000-0002-8529-9595)

CONFIDENTIALITY NOTICE
This document contains confidential information intended solely for the recipient and may be legally privileged

epidemiological characteristics of the two groups (CPS-followed and non-CPS-followed patients) are different.

- **KPI subvariations:** subvariations can be created by considering risk adjustments due to the hospital's epidemiology and specialty (see examples below). However, the use of KPI subvariations depends on an adequate definition of the limiting concepts introduced both in the numerator and denominator. Also, they should be interpreted as “complementary” to the overall assessment of the Clinical Pharmacy Service.
    - Examples:
        - Length of stay in patients admitted to Intensive Care Units (ICUs) *(specific ward risk adjustment)*
        - Length of stay of pediatric patients *(specific age risk adjustment)*
        - Length of stay of oncologic patients *(specific disease risk adjustment)*
        - Saved days of length of stay *(specific disease risk adjustment)*
            - This metric should define an ideal length of stay for each diagnosis, and then calculate the difference in length of stay between "expected" and "observed" LOS. If the difference is negative (observed LOS < expected LOS), there was a saving in length of stay; if the difference is positive (observed LOS > expected LOS), there was an increase in relation to the ideal time. However, as with all LOS measures, its result must be evaluated fully considering the assistance provided, not being attributed solely to the actions of the CPS.

CONFIDENTIAL

The Clinical Pharmacy KPI research team

Msc. Pharm. Lucas Magedanz (https://orcid.org/0000-0001-9812-2783)
Dr.ª Pharm. Dayani Galato (https://orcid.org/0000-0002-9295-8018)
Dr. Fernando Fernandez-Llimos (https://orcid.org/0000-0002-8529-9595)

CONFIDENTIALITY NOTICE
This document contains confidential information intended solely for the recipient and may be legally privileged

Purpose: This KPI aims to evaluate the medication goal achievement rate in patients followed by the hospital's Clinical Pharmacy Service (CPS).

Calculation formula:

$$\textbf{Medication goal achievement rate} = \left\{ \frac{\text{number of medications that achieved the planned goal in patients followed by the Clinical Pharmacy Service (CPS), in a certain period of time}}{\text{total number of drug therapies followed by the CPS in the hospital, in the same period of time}} \times 1000 \right\} = \textbf{Incidence rate of drug therapies that achieved the planned therapeutic goal per 1,000 therapies followed by the CPS, in the hospital in a certain period of time}$$

Key concepts:

- **Medication goal:** desired effect or objective of a specific medication regimen. It represents the intended purpose for which a medication is prescribed or administered to a patient. Medication goals are established based on the medical condition being treated, the patient's individual characteristics and needs, and the desired clinical outcomes. **The medication goal comprises objective/quantitative and subjective/qualitative outcomes, and must be previously defined by the healthcare team and clinical protocols considering the patient's individual needs.** Examples of medication goals include:
    - Achieving and maintaining blood pressure within a target range to reduce the risk of cardiovascular events in patients with hypertension.
    - Controlling blood glucose levels to prevent complications and improve quality of life in patients with diabetes.
    - Achieving viral suppression to improve immune function and reduce the risk of disease progression in patients with HIV/AIDS.
    - Pain control in patients with different clinical conditions.
    - Maintenance of sedation, without physiological impairment or manifestation of adverse events, in patients in induced coma.

Rational:

- This KPI reflects if patients achieve their medications' intended therapeutic outcomes. By tracking this KPI, healthcare providers can assess the effectiveness of medication management practices in meeting treatment goals, controlling symptoms, and improving overall patient health and well-being.

The Clinical Pharmacy KPI research team

Msc. Pharm. Lucas Magedanz (https://orcid.org/0000-0001-9812-2783)
Dr.ª Pharm. Dayani Galato (https://orcid.org/0000-0002-9295-8018)
Dr. Fernando Fernandez-Llimos (https://orcid.org/0000-0002-8529-9595)

CONFIDENTIALITY NOTICE
This document contains confidential information intended solely for the recipient and may be legally privileged

    - Important note: goals encompass both physiological parameters (e.g., blood pressure, cholesterol, etc.) as well as pain/function metrics (e.g., management of CHF, now able to walk X number of meters/flights of stairs).
- This KPI considers all therapies monitored by the CPS that may have an established therapeutic target (which is not always applicable to all therapies).

Operational considerations:

- **Category of the indicator:** clinic outcome.
- **Frequency of measure:** monthly.
- **KPI monitoring:** it is expected that the monthly results measured by the KPI will be compared with each other (longitudinal monitoring).
    - An alternative approach considers evaluating the same indicator in "patients not followed by the CPS", thus creating a cohort comparator (transversal monitoring). This approach, however, must be carefully evaluated, especially when the epidemiological characteristics of the two groups (CPS-followed and non-CPS-followed patients) are different.
- **Polarity of the measure:** the more, the better.
- **KPI subvariations:** subvariations can be created by considering risk adjustments due to the hospital's epidemiology and specialty (see examples below). However, the use of KPI subvariations depends on an adequate definition of the limiting concepts introduced both in the numerator and denominator. Also, they should be interpreted as "complementary" to the overall assessment of the Clinical Pharmacy Service.
    - In this specific case, the subvariations of this KPI are those in which separate measures are calculated for each monitored therapy (e.g., anticoagulation therapy, anti-infective treatment, etc.) However, it is essential to aggregate all therapies into a general (primary) indicator for an overview.
    - Examples:
        - Drug therapeutic goal rate in patients admitted to Intensive Care Units (ICUs) *(specific ward risk adjustment)*
        - Drug therapeutic goal rate in pediatric patients *(specific age risk adjustment)*
        - Drug therapeutic goal rate in oncologic patients *(specific disease risk adjustment)*

CONFIDENTIAL

The Clinical Pharmacy KPI research team

Msc. Pharm. Lucas Magedanz (https://orcid.org/0000-0001-9812-2783)
Dr.ª Pharm. Dayani Galato (https://orcid.org/0000-0002-9295-8018)
Dr. Fernando Fernandez-Llimos (https://orcid.org/0000-0002-8529-9595)

CONFIDENTIALITY NOTICE
This document contains confidential information intended solely for the recipient and may be legally privileged

Purpose: This KPI aims to evaluate canceled planned care (e.g., canceled surgery, canceled discharge from ICU or hospital, etc.) due to suboptimal medication management in patients followed by the hospital's Clinical Pharmacy Service (CPS).

Calculation formula:

$$\text{Medication-related canceled planned care rate} = \left\{ \frac{\text{number of canceled planned cares due to suboptimal medication management in patients followed by the Clinical Pharmacy Service (CPS), in a certain period of time}}{\text{total number of patient days followed by the CPS in the hospital, in the same period of time}} \times 1000 \right\} = \text{Incidence rate of canceled planned care per 1,000 patient days followed by the CPS, in the hospital in a certain period of time}$$

Key concepts:

- **Canceled planned care due to suboptimal medication management:** medical or healthcare services scheduled or anticipated to be provided to a patient but subsequently canceled or postponed for various reasons. In our case, we focus on cancellations due to suboptimal medicine management, which can include:
    - Medication Non-adherence or Inappropriate Medication Use
    - Drug-Drug Interactions
    - Suboptimal Dosage Adjustment
    - Lack of Medication Monitoring
    - Adverse Drug Events (ADEs)
    - Medication Errors

Rational:

- Suboptimal medication management can disrupt planned care schedules, leading to inefficiencies in resource utilization, workflow disruptions, and delays in patient care delivery. By this KPI, healthcare organizations can identify bottlenecks, streamline medication-related processes, and optimize operational efficiency to ensure timely and effective delivery of care to patients.

Operational considerations:

- **Category of the indicator:** clinic outcome.
- **Frequency of measure:** monthly.
- **Polarity of the measure:** the less, the better.

CONFIDENTIAL

The Clinical Pharmacy KPI research team

Msc. Pharm. Lucas Magedanz (https://orcid.org/0000-0001-9812-2783)
Dr.ª Pharm. Dayani Galato (https://orcid.org/0000-0002-9295-8018)
Dr. Fernando Fernandez-Llimos (https://orcid.org/0000-0002-8529-9595)

CONFIDENTIALITY NOTICE
This document contains confidential information intended solely for the recipient and may be legally privileged

- **KPI monitoring:** it is expected that the monthly results measured by the KPI will be compared with each other (longitudinal monitoring).
    - An alternative approach considers evaluating the same indicator in "patients not followed by the CPS", thus creating a cohort comparator (transversal monitoring). This approach, however, must be carefully evaluated, especially when the epidemiological characteristics of the two groups (CPS-followed and non-CPS-followed patients) are different.
- **KPI subvariations:** subvariations can be created by considering risk adjustments due to the hospital's epidemiology and specialty (see examples below). However, the use of KPI subvariations depends on an adequate definition of the limiting concepts introduced both in the numerator and denominator. Also, they should be interpreted as "complementary" to the overall assessment of the Clinical Pharmacy Service.
    - Examples:
        - Medication-related cancellation of planned care rate in patients admitted to Intensive Care Units (ICUs) *(specific ward risk adjustment)*
        - Medication-related cancellation of planned care rate in pediatric patients *(specific age risk adjustment)*
        - Medication-related cancellation of planned care rate in oncologic patients *(specific disease risk adjustment)*

CONFIDENTIAL

The Clinical Pharmacy KPI research team

Msc. Pharm. Lucas Magedanz (https://orcid.org/0000-0001-9812-2783)
Dr.ª Pharm. Dayani Galato (https://orcid.org/0000-0002-9295-8018)
Dr. Fernando Fernandez-Llimos (https://orcid.org/0000-0002-8529-9595)

CONFIDENTIALITY NOTICE
This document contains confidential information intended solely for the recipient and may be legally privileged

Purpose: This KPI aims to evaluate the rate of all-cause unplanned readmissions in hospital wards within 30 days of a hospital discharge, in patients followed by the hospital's Clinical Pharmacy Service (CPS).

Calculation formula:

$$\textbf{All-cause unplanned 30-day readmission rate} = \left\{ \frac{\text{number of all-cause unplanned readmissions in hospital ward that occurred up to 30 days after hospital discharge in patients followed by the Clinical Pharmacy Service (CPS), in a certain period of time}}{\text{number of patients discharged from the hospital, in the same period of time}} \times 1000 \right\} = \textbf{Incidence rate of all-cause unplanned 30-day readmissions in hospital ward per 1,000 patients followed by the CPS, in the hospital in a certain period of time}$$

Key concepts:

- **Unplanned readmission:** the occurrence of an unplanned patient returning to the hospital for inpatient care within a specified period after being discharged from a previous hospitalization.

Rational:

- Effective care transitions from hospital to home or other care settings are essential for preventing unplanned readmissions. Monitoring the 30-day readmission rate as a KPI allows healthcare organizations to evaluate the effectiveness of care transition processes, identify barriers or gaps in communication, and implement strategies to facilitate seamless transitions of care and reduce the risk of hospital readmission.

Operational considerations:

- **Category of the indicator:** clinic outcome.
- **Frequency of measure:** monthly.
- **Polarity of the measure:** the less, the better.
  - Important note: Although we recognize that CPS is not the only preponderant factor in unplanned hospital readmissions, studies have shown that CPS can modify this variable.
- **KPI monitoring:** it is expected that the monthly results measured by the KPI will be compared with each other (longitudinal monitoring).
  - An alternative approach considers evaluating the same indicator in “patients not followed by the CPS”, thus creating a cohort comparator (transversal monitoring). This approach, however, must be carefully evaluated, especially when the

CONFIDENTIAL

The Clinical Pharmacy KPI research team

Msc. Pharm. Lucas Magedanz (https://orcid.org/0000-0001-9812-2783)
Dr.ª Pharm. Dayani Galato (https://orcid.org/0000-0002-9295-8018)
Dr. Fernando Fernandez-Llimos (https://orcid.org/0000-0002-8529-9595)

CONFIDENTIALITY NOTICE
This document contains confidential information intended solely for the recipient and may be legally privileged

epidemiological characteristics of the two groups (CPS-followed and non-CPS-followed patients) are different.

- **KPI subvariations:** subvariations can be created by considering risk adjustments due to the hospital's epidemiology and specialty (see examples below). However, the use of KPI subvariations depends on an adequate definition of the limiting concepts introduced both in the numerator and denominator. Also, they should be interpreted as “complementary” to the overall assessment of the Clinical Pharmacy Service.
    - Examples:
        - All-cause unplanned 30-day readmission rate for the same reason that last admission (specific ward risk adjustment)
            - This metric requires a medical panel to judge each readmission to ensure that the new admission is related to the previous admission.
        - All-cause unplanned 30-day readmission rate in patients admitted to Intensive Care Units (ICUs) *(specific ward risk adjustment)*
        - All-cause unplanned 30-day readmission rate in pediatric patients *(specific age risk adjustment)*
        - All-cause unplanned 30-day readmission rate in oncologic patients *(specific disease risk adjustment)*
        - Medication-related unplanned 30-day readmission rate *(specific cause risk adjustment)*
        - All-cause unplanned 90-day readmission rate *(longest period variation)*

CONFIDENTIAL

The Clinical Pharmacy KPI research team

Msc. Pharm. Lucas Magedanz (https://orcid.org/0000-0001-9812-2783)
Dr.ª Pharm. Dayani Galato (https://orcid.org/0000-0002-9295-8018)
Dr. Fernando Fernandez-Llimos (https://orcid.org/0000-0002-8529-9595)

CONFIDENTIALITY NOTICE
This document contains confidential information intended solely for the recipient and may be legally privileged

Purpose: This KPI aims to evaluate the all-cause mortality rate in patients followed by the hospital's Clinical Pharmacy Service (CPS).

Calculation formula:

$$\text{All-cause mortality rate} = \left\{ \frac{\text{number of all-cause deaths that occurred in patients followed by the Clinical Pharmacy Service (CPS), in a certain period of time}}{\text{total number of patients admitted to the hospital and followed by the CPS, in the same period of time}} \times 1000 \right\} = \text{Incidence rate of deaths per 1,000 admissions in patients followed by the CPS, in the hospital in a certain period of time}$$

Key concepts:

- **Mortality:** death of patients that occurs within a healthcare facility, such as a hospital, during medical treatment or while under the care of healthcare professionals.

Rational:

- This KPI reflects the ultimate result of medical interventions, treatments, and healthcare delivery processes. By tracking mortality rates as a KPI, healthcare organizations can assess the impact of their practices on patient outcomes, identify areas for improvement, and implement targeted interventions to enhance patient survival and overall health.

Operational considerations:

- **Category of the indicator:** clinic outcome.
- **Frequency of measure:** monthly.
- **Polarity of the measure:** the less, the better.
  - Important note: Although we recognize that CPS is not the only preponderant factor in mortality, studies have shown that CPS can modify this variable.
- **KPI monitoring:** it is expected that the monthly results measured by the KPI will be compared with each other (longitudinal monitoring).
  - An alternative approach considers evaluating the same indicator in "patients not followed by the CPS", thus creating a cohort comparator (transversal monitoring). This approach, however, must be carefully evaluated, especially when the epidemiological characteristics of the two groups (CPS-followed and non-CPS-followed patients) are different.

The Clinical Pharmacy KPI research team

Msc. Pharm. Lucas Magedanz (https://orcid.org/0000-0001-9812-2783)
Dr.ª Pharm. Dayani Galato (https://orcid.org/0000-0002-9295-8018)
Dr. Fernando Fernandez-Llimos (https://orcid.org/0000-0002-8529-9595)

CONFIDENTIALITY NOTICE
This document contains confidential information intended solely for the recipient and may be legally privileged

CONFIDENTIAL

- **KPI subvariations:** subvariations can be created by considering risk adjustments due to the hospital's epidemiology and specialty (see examples below). However, the use of KPI subvariations depends on an adequate definition of the limiting concepts introduced both in the numerator and denominator. Also, they should be interpreted as "complementary" to the overall assessment of the Clinical Pharmacy Service.
  - Examples:
    - All-cause mortality rate in patients admitted to Intensive Care Units (ICUs) *(specific ward risk adjustment)*
    - All-cause mortality rate in pediatric patients (*specific age risk adjustment)*
    - All-cause mortality rate in oncologic patients *(specific disease risk adjustment)*
    - Medication-related mortality rate *(specific cause risk adjustment)*

CONFIDENTIAL

The Clinical Pharmacy KPI research team

Msc. Pharm. Lucas Magedanz (https://orcid.org/0000-0001-9812-2783)
Dr.ª Pharm. Dayani Galato (https://orcid.org/0000-0002-9295-8018)
Dr. Fernando Fernandez-Llimos (https://orcid.org/0000-0002-8529-9595)

CONFIDENTIALITY NOTICE
This document contains confidential information intended solely for the recipient and may be legally privileged

Purpose: This KPI aims to evaluate the rate of all-cause visits in emergency departments of hospitals within 30 days of a hospital discharge, in patients followed by the hospital's Clinical Pharmacy Service (CPS).

Calculation formula:

$$\text{All-cause 30-day emergency department visit rate} = \left\{ \frac{\text{number of all-cause emergency department visit that occurred up to 30 days after hospital discharge in patients followed by the Clinical Pharmacy Service (CPS), in a certain period of time}}{\text{number of patients discharged from the hospital, in the same period of time}} \times 1000 \right\} = \textbf{Incidence rate of all-cause unplanned 30-day emergency department visit per 1,000 patients followed by the CPS, in the hospital in a certain period of time}$$

Key concepts:

- **Emergency department visit:** the occurrence of a patient returning to the hospital's emergency department after being discharged from a previous hospitalization.
    - This KPI considers only "unplanned" emergency visits (it does not comprehend ambulatory visits/consults).

Rational:

- Effective care transitions from hospital to home or other care settings are essential for preventing unplanned emergency department visits. Monitoring the 30-day emergency department visit rate as a KPI allows healthcare organizations to evaluate the effectiveness of care transition processes, identify barriers or gaps in communication, and implement strategies to facilitate seamless transitions of care and reduce the risk of readmission.

Operational considerations:

- **Category of the indicator:** clinic outcome.
- **Frequency of measure:** monthly.
- **Polarity of the measure:** the less, the better.
    - Important note: Although we recognize that CPS is not the only preponderant factor in unplanned emergency department visits, studies have shown that CPS can modify this variable.

The Clinical Pharmacy KPI research team

Msc. Pharm. Lucas Magedanz (https://orcid.org/0000-0001-9812-2783)
Dr.ª Pharm. Dayani Galato (https://orcid.org/0000-0002-9295-8018)
Dr. Fernando Fernandez-Llimos (https://orcid.org/0000-0002-8529-9595)

CONFIDENTIALITY NOTICE
This document contains confidential information intended solely for the recipient and may be legally privileged

- **KPI monitoring:** it is expected that the monthly results measured by the KPI will be compared with each other (longitudinal monitoring).
  - An alternative approach considers evaluating the same indicator in "patients not followed by the CPS", thus creating a cohort comparator (transversal monitoring). This approach, however, must be carefully evaluated, especially when the epidemiological characteristics of the two groups (CPS-followed and non-CPS-followed patients) are different.
- **KPI subvariations:** subvariations can be created by considering risk adjustments due to the hospital's epidemiology and specialty (see examples below). However, the use of KPI subvariations depends on an adequate definition of the limiting concepts introduced both in the numerator and denominator. Also, they should be interpreted as "complementary" to the overall assessment of the Clinical Pharmacy Service.
  - Examples:
    - All-cause 30-day emergency visit rate for the same reason that last admission (specific ward risk adjustment)
      - This metric requires a medical panel to judge each readmission to ensure that the new admission is related to the previous admission.
    - All-cause unplanned 30-day emergency department visit rate in patients admitted to Intensive Care Units (ICUs) *(specific ward risk adjustment)*
    - All-cause unplanned 30-day emergency department visit rate in pediatric patients *(specific age risk adjustment)*
    - All-cause unplanned 30-day emergency department visit rate in oncologic patients *(specific disease risk adjustment)*
    - Medication-related unplanned 30-day emergency department visit rate *(specific cause risk adjustment)*
    - All-cause 90-day emergency visit rate *(longest period variation)*

CONFIDENTIAL

The Clinical Pharmacy KPI research team

Msc. Pharm. Lucas Magedanz (https://orcid.org/0000-0001-9812-2783)
Dr.ª Pharm. Dayani Galato (https://orcid.org/0000-0002-9295-8018)
Dr. Fernando Fernandez-Llimos (https://orcid.org/0000-0002-8529-9595)

CONFIDENTIALITY NOTICE
This document contains confidential information intended solely for the recipient and may be legally privileged

KPI information sheet

Clinical Pharmacy KPI: **Average Clinical Pharmacy Service revenue generated**

Version: 03	Date: April / 2024	Page 1/2

Purpose: This KPI aims to calculate the average revenue generated by the hospital's Clinical Pharmacy Service (CPS).

Calculation formula:

$$\text{Average Clinical Pharmacy Service revenue generated} = \left\{ \frac{\text{Sum of amounts billed/invoiced by the Clinical Pharmacy Service (CPS) in the hospital, in a certain period of time}}{\text{total number of patient days followed by the CPS in the hospital, in the same period of time}} \times 1000 \right\} = \text{Estimated average revenue generated (in US dollars or local currency) per 1,000 patient days followed by the CPS, in the hospital in a certain period of time}$$

Key concepts:

- **Clinical pharmacist billing/invoice:** Clinical pharmacist billing/invoice: income generated by Clinical Pharmacy Services (CPSs) provided within the healthcare facility, which may be billable to third-party payers. Examples of clinical pharmacy services include:
  - medication reviews;
  - patient education/counseling;
  - medication reconciliation;
  - medication therapy optimization;

Rational:

- Tracking the number of revenue generated provides insight into the organization's financial health. As hospitals rely on revenue from patient services, including billable procedures, treatments, and consultations, monitoring billing invoices helps ensure a steady flow of income to support operations, investments, and sustainability.
- We recognize that direct remuneration for pharmaceutical services is not yet a reality in all health systems, public or private, worldwide. However, this is already a reality in some locations, and it could be a compensation method that could grow with the development of pharmaceutical assistance.

Operational considerations:

- **Category of the indicator:** economic outcome.
- **Frequency of measure:** monthly.
- **Polarity of the measure:** the more, the better.

CONFIDENTIAL

The Clinical Pharmacy KPI research team

Msc. Pharm. Lucas Magedanz (https://orcid.org/0000-0001-9812-2783)
Dr.ª Pharm. Dayani Galato (https://orcid.org/0000-0002-9295-8018)
Dr. Fernando Fernandez-Llimos (https://orcid.org/0000-0002-8529-9595)

CONFIDENTIALITY NOTICE
This document contains confidential information intended solely for the recipient and may be legally privileged

- **KPI monitoring:** it is expected that the monthly results measured by the KPI will be compared with each other (longitudinal monitoring).
- **KPI subvariations:** subvariations can be created by considering risk adjustments due to the hospital's epidemiology and specialty (see examples below). However, the use of KPI subvariations depends on an adequate definition of the limiting concepts introduced both in the numerator and denominator. Also, they should be interpreted as "complementary" to the overall assessment of the Clinical Pharmacy Service.
    - Examples:
        - Clinical Pharmacy Service revenue generated in patients admitted to Intensive Care Units (ICUs) *(specific ward risk adjustment)*
        - Clinical Pharmacy Service revenue generated in pediatric patients *(specific age risk adjustment)*
        - Clinical Pharmacy Service revenue generated in oncologic patients *(specific disease risk adjustment)*
        - Clinical Pharmacy Service revenue generated from medication reconciliation activities *(specific service/activity risk adjustment)*

CONFIDENTIAL

The Clinical Pharmacy KPI research team

Msc. Pharm. Lucas Magedanz (https://orcid.org/0000-0001-9812-2783)
Dr.ª Pharm. Dayani Galato (https://orcid.org/0000-0002-9295-8018)
Dr. Fernando Fernandez-Llimos (https://orcid.org/0000-0002-8529-9595)

CONFIDENTIALITY NOTICE
This document contains confidential information intended solely for the recipient and may be legally privileged

Purpose: This KPI aims to calculate the difference in cost between a given medication ("original medication") and an "optimized treatment" (including changes in drug, dose, regime, and even discontinuation of the medication) suggested by the hospital's Clinical Pharmacy Service (CPS) and accepted by the healthcare team.

Calculation formula:

Total optimized treatment cost difference = { $\sum$ (Sum of all "individual" direct treatment cost cases saved by CPS, in a certain period of time) [(cost of treatment day (in US dollars or local currency) of the optimized treatment suggested by the CPS and ACCEPTED by the healthcare team **−** cost of treatment day (in US dollars or local currency) of the given medication (original)) **x** number of days that the original medication was replaced by the optimized treatment] } **=** **Estimated total costs differences (in US dollars or local currency) from all treatments optimized by the CPS, in the hospital in a certain period of time**

Key concepts:

- **Cost of treatment day:** cost of treatment in 1 day. If a treatment unit can be used for more than one day (e.g., a bottle of liquid medicine, in which the same bottle is used by the patient for several days), the cost must be divided by the proportion of the part to be used. In the case of drugs/medicines, the treatment day is estimated as follows:
  - **Cost of treatment day = (drug's dose cost) x (number of doses used per day)**

- **Optimized treatment suggested by a clinical pharmacist:** recommendation made by the pharmacist regarding the selection, dosing, administration, or monitoring of medications as part of a patient's treatment plan. It's performed to optimize medication therapy and ensure safe and effective drug use by collaborating with healthcare teams. Examples:
  - Medication substitution
  - Medication discontinuation (deprescribing)
  - Dose adjustment
  - Formulation changes (e.g., switch endovenous to oral route of administration, capsule to liquid to administration via enteral tube, etc.)

Rational:

- This KPI quantifies the financial differences achieved by optimizing treatment protocols, medication regimens, and healthcare practices. By monitoring this KPI,

The Clinical Pharmacy KPI research team

Msc. Pharm. Lucas Magedanz (https://orcid.org/0000-0001-9812-2783)
Dr.ª Pharm. Dayani Galato (https://orcid.org/0000-0002-9295-8018)
Dr. Fernando Fernandez-Llimos (https://orcid.org/0000-0002-8529-9595)

CONFIDENTIALITY NOTICE
This document contains confidential information intended solely for the recipient and may be legally privileged

CONFIDENTIAL

healthcare organizations can understand the direct impact of the CPS on financial hospital measures.

- **KPI result interpretation:**
    - A "**positive**" result means that the optimized treatment had a greater financial cost compared to the original treatment.
    - A "**negative**" result demonstrates direct cost savings.
    - **Important note: It is essential to understand that this KPI only aims to show the financial impact of the CPS's interventions, which must always be guided by improving treatment, considering the individual's particular needs, and the precepts of rational use of medicines.**
        - The World Health Organization (WHO) defines rational use of medicine as "patients receiving medications appropriate to their clinical needs, in doses that meet their own individual requirements, for an adequate period of time, and at the lowest cost to them and their community".

Operational considerations:

- **Category of the indicator:** economic outcome.
- **Frequency of measure:** monthly.
- **Polarity of the measure:** the more, the better.
- **KPI monitoring:** it is expected that the monthly results measured by the KPI will be compared with each other (longitudinal monitoring).
- **KPI subvariations:** subvariations can be created by considering risk adjustments due to the hospital's epidemiology and specialty (see examples below). However, the use of KPI subvariations depends on an adequate definition of the limiting concepts introduced both in the numerator and denominator. Also, they should be interpreted as "complementary" to the overall assessment of the Clinical Pharmacy Service.
    - Examples:
        - Total optimized treatment cost difference in patients admitted to Intensive Care Units (ICUs) *(specific ward risk adjustment)*
        - Total optimized treatment cost difference in pediatric patients *(specific age risk adjustment)*
        - Total optimized treatment cost difference in oncologic patients *(specific disease risk adjustment)*

CONFIDENTIAL

The Clinical Pharmacy KPI research team

Msc. Pharm. Lucas Magedanz (https://orcid.org/0000-0001-9812-2783)
Dr.ª Pharm. Dayani Galato (https://orcid.org/0000-0002-9295-8018)
Dr. Fernando Fernandez-Llimos (https://orcid.org/0000-0002-8529-9595)

CONFIDENTIALITY NOTICE
This document contains confidential information intended solely for the recipient and may be legally privileged

Purpose: This KPI aims to calculate the difference in cost between a given medication ("original medication") and an "optimized treatment" (including changes in drug, dose, regime, and even discontinuation of the medication) suggested by the hospital's Clinical Pharmacy Service (CPS) and accepted by the healthcare team.

Calculation formula:

$$\textbf{Average optimized treatment cost difference} = \sum \left[\frac{\left(\text{cost of treatment day (in US dollars or local currency) of the optimized treatment suggested by the CPS and ACCEPTED by the healthcare team} - \text{cost of treatment day (in US dollars or local currency) of the given medication (original)} \right) \times \text{number of days that the original medication was replaced by the optimized treatment}}{\text{number of optimized therapeutic options suggested by the CPS and ACCEPTED by the healthcare team in the hospital, in the same period of time}} \right] = \textbf{Average of the estimated total costs differences (in US dollars or local currency) from the treatments optimized by the CPS, in the hospital in a certain period of time}$$

$\sum$: Sum of all "individual" direct drug cost cases saved by CPS, in a certain period of time

Key concepts:

- **Cost of treatment day:** cost of treatment in 1 day. If a treatment unit can be used for more than one day (e.g., a bottle of liquid medicine, in which the same bottle is used by the patient for several days), the cost must be divided by the proportion of the part to be used. In the case of drugs/medicines, the treatment day is estimated as follows:
  - Cost of treatment day = (drug's dose cost) x (number of doses used per day)
- **Optimized treatment suggested by a clinical pharmacist:** recommendation made by the pharmacist regarding the selection, dosing, administration, or monitoring of medications as part of a patient's treatment plan. It's performed to optimize medication therapy and ensure safe and effective drug use by collaborating with healthcare teams. Examples:
  - Medication substitution
  - Medication discontinuation (deprescribing)
  - Dose adjustment
  - Formulation changes (e.g., switch endovenous to oral route of administration, capsule to liquid to administration via enteral tube, etc.)

Rational:

- This KPI quantifies the financial differences by optimizing treatment protocols, medication regimens, and healthcare practices. By monitoring this KPI, healthcare

The Clinical Pharmacy KPI research team

Msc. Pharm. Lucas Magedanz (https://orcid.org/0000-0001-9812-2783)
Dr.ª Pharm. Dayani Galato (https://orcid.org/0000-0002-9295-8018)
Dr. Fernando Fernandez-Llimos (https://orcid.org/0000-0002-8529-9595)

CONFIDENTIALITY NOTICE
This document contains confidential information intended solely for the recipient and may be legally privileged

organizations can understand the direct impact of the CPS on financial hospital measures.

- The average cost differences present an average value for therapeutic optimization, which can be used to project savings prospects over a long-term horizon and used by the hospital administration in its financial planning.
- **KPI result interpretation:**
  - A "**positive**" result means that the optimized treatment had a greater financial cost compared to the original treatment.
  - A "**negative**" result demonstrates direct cost savings.
  - **Important note: It is essential to understand that this KPI only aims to show the financial impact of the CPS's interventions, which must always be guided by improving treatment, considering the individual's particular needs, and the precepts of rational use of medicines.**
    - The World Health Organization (WHO) defines rational use of medicine as "patients receiving medications appropriate to their clinical needs, in doses that meet their own individual requirements, for an adequate period of time, and at the lowest cost to them and their community".

Operational considerations:

- **Category of the indicator:** economic outcome.
- **Frequency of measure:** monthly.
- **Polarity of the measure:** the more, the better.
- **KPI monitoring:** it is expected that the monthly results measured by the KPI will be compared with each other (longitudinal monitoring).
- **KPI subvariations:** subvariations can be created by considering risk adjustments due to the hospital's epidemiology and specialty (see examples below). However, the use of KPI subvariations depends on an adequate definition of the limiting concepts introduced both in the numerator and denominator. Also, they should be interpreted as “complementary” to the overall assessment of the Clinical Pharmacy Service.
  - Examples:
    - Average optimized treatment cost difference in patients admitted to Intensive Care Units (ICUs) *(specific ward risk adjustment)*
    - Average optimized treatment cost difference in pediatric patients *(specific age risk adjustment)*
    - Average optimized treatment cost difference in oncologic patients *(specific disease risk adjustment)*
    - Median optimized treatment difference *(alternative evaluation)*
      - *This metric changes the average calculation to a median*

The Clinical Pharmacy KPI research team

Msc. Pharm. Lucas Magedanz (https://orcid.org/0000-0001-9812-2783)
Dr.ª Pharm. Dayani Galato (https://orcid.org/0000-0002-9295-8018)
Dr. Fernando Fernandez-Llimos (https://orcid.org/0000-0002-8529-9595)

CONFIDENTIALITY NOTICE
This document contains confidential information intended solely for the recipient and may be legally privileged

CONFIDENTIAL

CONFIDENTIAL

The Clinical Pharmacy KPI research team

Msc. Pharm. Lucas Magedanz (https://orcid.org/0000-0001-9812-2783)
Dr.ª Pharm. Dayani Galato (https://orcid.org/0000-0002-9295-8018)
Dr. Fernando Fernandez-Llimos (https://orcid.org/0000-0002-8529-9595)

CONFIDENTIALITY NOTICE
This document contains confidential information intended solely for the recipient and may be legally privileged

Clinical Pharmacy KPI: **Negative Reported Experience Measures (PREMs) rate**

Purpose: This KPI aims to evaluate the frequency of negative Patient Reported Experience Measures (PREMs) related to Clinical Pharmacy Service (CPS).

Calculation formula:

$$\text{Negative Patient Reported Experience Measures (PREMs) rate} = \left\{ \frac{\text{Total number NEGATIVE Patient Reported Experience Measures (PREMs) related to the Clinical Pharmacy Service (CPS) in the hospital, in a certain period of time}}{\text{Total number of PREMs (positive + negative) related to the CPS in the hospital, in the same period of time}} \times 1000 \right\} = \text{Incidence rate of NEGATIVE PREMs per 1,000 PREMs related to the CPS, in the hospital, in a certain period of time}$$

Key concepts:

- **Patient Reported Experience Measures (PREMs):** quantitative or qualitative measures that focus on gathering feedback directly from patients regarding various aspects of their healthcare experience, including communication with healthcare providers, quality of care, accessibility of services, and overall satisfaction.

Rational:

- This KPI focuses on capturing patients' positive/negative perspectives, experiences, and satisfaction with healthcare services. By incorporating PREMs as a KPI, healthcare organizations demonstrate their commitment to patient-centered care and prioritize the voices and needs of patients in evaluating and improving service delivery.
- The choice to emphasize negative PREMs is because negative experiences tend to be reported more frequently.
- In case the institution combines the PREMs into Likert scales, the **negative** PREMS would weigh from "strongly disagree", "disagree", and "neutral", and the **positive** PREMs would be just "agree" and "strongly agree".

Operational considerations:

- **Category of the indicator:** humanistic outcome.
- **Frequency of measure:** monthly.
- **Polarity of the measure:** the less, the better.

The Clinical Pharmacy KPI research team

Msc. Pharm. Lucas Magedanz (https://orcid.org/0000-0001-9812-2783)
Dr.ª Pharm. Dayani Galato (https://orcid.org/0000-0002-9295-8018)
Dr. Fernando Fernandez-Llimos (https://orcid.org/0000-0002-8529-9595)

CONFIDENTIALITY NOTICE
This document contains confidential information intended solely for the recipient and may be legally privileged

CONFIDEN

- **KPI monitoring:** it is expected that the monthly results measured by the KPI will be compared with each other (longitudinal monitoring).
- **KPI subvariations:** subvariations can be created by considering risk adjustments due to the hospital's epidemiology and specialty (see examples below). However, the use of KPI subvariations depends on an adequate definition of the limiting concepts introduced both in the numerator and denominator. Also, they should be interpreted as "complementary" to the overall assessment of the Clinical Pharmacy Service.
    - Examples:
        - Negative Reported Experience Measures (PREMs) rate in patients admitted to Intensive Care Units (ICUs) *(specific ward risk adjustment)*
        - Negative Reported Experience Measures (PREMs) rate in pediatric patients *(specific age risk adjustment)*
        - Negative Reported Experience Measures (PREMs) rate in oncologic patients *(specific disease risk adjustment)*

CONFIDENTIAL

The Clinical Pharmacy KPI research team

Msc. Pharm. Lucas Magedanz (https://orcid.org/0000-0001-9812-2783)
Dr.ª Pharm. Dayani Galato (https://orcid.org/0000-0002-9295-8018)
Dr. Fernando Fernandez-Llimos (https://orcid.org/0000-0002-8529-9595)

CONFIDENTIALITY NOTICE
This document contains confidential information intended solely for the recipient and may be legally privileged

Purpose: This KPI aims to evaluate the results of satisfaction surveys applied to patients followed by the hospital's Clinical Pharmacy Service (CPS).

Calculation formula:

Patient satisfaction survey results = { Results of satisfaction surveys collected from patients followed by the Clinical Pharmacy Service (CPS) in the hospital, in a certain period of time } = **Satisfaction perception in patients followed by the CPS in the hospital, in a certain period of time**

Key concepts:

- **Patient satisfaction:** subjective evaluation and perception of healthcare services and patient experiences during interactions with the hospital and its staff. It encompasses the extent to which patients' expectations, preferences, and needs are met and their overall level of contentment, comfort, and satisfaction with the care received.

Rational:

- This KPI reflects how healthcare services meet patients' needs, preferences, and expectations. By incorporating patient satisfaction as a KPI, healthcare organizations demonstrate their commitment to patient-centered care and prioritize the voices and experiences of patients in evaluating and improving service delivery.
- The literature presents several models of instruments for assessing patient satisfaction. In general, these models are extensive and complex to be applied to all patients in the hospital routine. Furthermore, quantifying the contribution of each professional specialty in the context of collaborative interprofessional care is complex. In this KPI proposal, **we suggest that the evaluation of "patient satisfaction" considers the institution's evaluation routine**, thus analyzing the final result.
- In case the institution evaluates the satisfaction of the patient into Likert scales, the **negative** PREMS would weigh from "strongly disagree", "disagree", and "neutral", and the **positive** PREMs would be just "agree" and "strongly agree".

Operational considerations:

- **Category of the indicator:** humanistic outcome.
- **Frequency of measure:** monthly.

The Clinical Pharmacy KPI research team

Msc. Pharm. Lucas Magedanz (https://orcid.org/0000-0001-9812-2783)
Dr.ª Pharm. Dayani Galato (https://orcid.org/0000-0002-9295-8018)
Dr. Fernando Fernandez-Llimos (https://orcid.org/0000-0002-8529-9595)

CONFIDENTIALITY NOTICE
This document contains confidential information intended solely for the recipient and may be legally privileged

CONFIDENTIAL

- **Polarity of the measure:** the more, the better.
- **KPI monitoring:** it is expected that the monthly results measured by the KPI will be compared with each other (longitudinal monitoring).
- **KPI subvariations:** subvariations can be created by considering risk adjustments due to the hospital's epidemiology and specialty (see examples below). However, the use of KPI subvariations depends on an adequate definition of the limiting concepts introduced both in the numerator and denominator. Also, they should be interpreted as "complementary" to the overall assessment of the Clinical Pharmacy Service.
    - Examples:
        - Patient satisfaction survey results in patients admitted to Intensive Care Units (ICUs) *(specific ward risk adjustment)*
        - Patient satisfaction survey results in pediatric patients *(specific age risk adjustment)*
        - Patient satisfaction survey results in oncologic patients *(specific disease risk adjustment)*

CONFIDENTIAL

The Clinical Pharmacy KPI research team

Msc. Pharm. Lucas Magedanz (https://orcid.org/0000-0001-9812-2783)
Dr.ª Pharm. Dayani Galato (https://orcid.org/0000-0002-9295-8018)
Dr. Fernando Fernandez-Llimos (https://orcid.org/0000-0002-8529-9595)

CONFIDENTIALITY NOTICE
This document contains confidential information intended solely for the recipient and may be legally privileged
